# Transcription factor EB (TFEB) is a new therapeutic target for Pompe disease

**DOI:** 10.1002/emmm.201202176

**Published:** 2013-04-18

**Authors:** Carmine Spampanato, Erin Feeney, Lishu Li, Monica Cardone, Jeong-A Lim, Fabio Annunziata, Hossein Zare, Roman Polishchuk, Rosa Puertollano, Giancarlo Parenti, Andrea Ballabio, Nina Raben

**Affiliations:** 1 Telethon Institute of Genetics and Medicine (TIGEM)Naples, Italy; 2 Department of Molecular and Human Genetics, Baylor College of MedicineHouston, TX, USA; 3 Jan and Dan Duncan Neurological Research Institute, Texas Children HospitalHouston, TX, USA; 4 Laboratory of Muscle Stem Cells and Gene Regulation, National Institute of Arthritis and Musculoskeletal and Skin Diseases, National Institutes of HealthBethesda, MD, USA; 5 Laboratory of Cell Biology, National Heart, Lung, and Blood Institute, National Institutes of HealthBethesda, MD, USA; 6 Department of Pediatrics, Federico II UniversityNaples, Italy

**Keywords:** acid alpha-glucosidase, autophagy, lysosomal storage, Pompe disease, TFEB

## Abstract

A recently proposed therapeutic approach for lysosomal storage disorders (LSDs) relies upon the ability of transcription factor EB (TFEB) to stimulate autophagy and induce lysosomal exocytosis leading to cellular clearance. This approach is particularly attractive in glycogen storage disease type II [a severe metabolic myopathy, Pompe disease (PD)] as the currently available therapy, replacement of the missing enzyme acid alpha-glucosidase, fails to reverse skeletal muscle pathology. PD, a paradigm for LSDs, is characterized by both lysosomal abnormality and dysfunctional autophagy. Here, we show that TFEB is a viable therapeutic target in PD: overexpression of TFEB in a new muscle cell culture system and in mouse models of the disease reduced glycogen load and lysosomal size, improved autophagosome processing, and alleviated excessive accumulation of autophagic vacuoles. Unexpectedly, the exocytosed vesicles were labelled with lysosomal and autophagosomal membrane markers, suggesting that TFEB induces exocytosis of autophagolysosomes. Furthermore, the effects of TFEB were almost abrogated in the setting of genetically suppressed autophagy, supporting the role of autophagy in TFEB-mediated cellular clearance.

## INTRODUCTION

Pompe disease (PD; OMIM 232300) is a severe metabolic myopathy caused by the deficiency of acid alpha-glucosidase (GAA; acid maltase, E.C.3.2.1.20), an enzyme responsible for breaking down glycogen to glucose within the acidic environment of lysosomes. The functional deficiency or complete absence of the enzyme results in accumulation of glycogen within this cellular compartment (Kroos et al, 2012; Van der Ploeg & Reuser, 2008). PD pathology is also characterized by secondary accumulation of autophagic debris (autophagic build-up), typically found in skeletal muscle fibres (Raben et al, 2007a, b, 2012). Although PD is a rare disorder (Martiniuk et al, 1998), it shares common pathological features with many other lysosomal storage disorders (LSDs), which encompass more than 60 nosological entities (Cox & Cachon-Gonzalez, 2012).

GAA deficiency is a systemic disorder – distended glycogen-filled lysosomes can be found in multiple tissues – but skeletal and cardiac muscles are particularly vulnerable to the accumulation of storage material. In the most serious infantile form, the disease manifests as profound weakness, hypertrophic cardiomyopathy, heart failure, feeding difficulties, respiratory infections and, if left untreated, causes death within the first year of life. In the attenuated phenotypes, characterized by later (childhood, juvenile or adult) onset, cardiac muscle is usually spared, but the illness remains a serious condition with progressive motor impairment, respiratory failure and premature death (Van der Ploeg & Reuser, 2008).

Enzyme replacement therapy (ERT) is now available for several LSDs including PD. Unfortunately, in many of these diseases the target tissues are not easily accessible to the replacement enzymes (Lachmann, 2011; Urbanelli et al, 2011). In PD, skeletal muscle – the major target for ERT – is particularly challenging to treat. Even with extremely high dosages of the drug, recombinant human GAA (rhGAA; alglucosidase alpha; Myozyme® and Lumizyme®, Genzyme Corporation, Framingham, MA), many patients experience limited clinical benefit or show signs of disease progression (Angelini & Semplicini, 2012; Kishnani et al, 2007; Schoser et al, 2008; Strothotte et al, 2010; Van den Hout et al, 2004; Van der Ploeg et al, 2010). Furthermore, the greatest success of ERT in PD – significantly improved survival of infants due to the restoration of cardiac function – comes at a price: many long-term survivors suffer from debilitating myopathy, often more severe than in late-onset cases.

Several factors contribute to the resistance of skeletal muscle to ERT. These include the sheer mass of muscle tissue, a relatively low density of the mannose-6-phosphate receptor that is responsible for the uptake of the enzyme, inefficient trafficking of the internalized enzyme to lysosomes, immune response to the recombinant enzyme in cross-reactive immunologic material (CRIM)-negative patients and the presence of large areas of autophagic build-up alongside expanded lysosomes (Banugaria et al, 2011; Funk et al, 1992; Kishnani et al, 2010; Koeberl et al, 2012; Raben et al, 2002, 2010, 2012).

Significant efforts are aimed at improving the current therapy, such as the development of “second generation” recombinant enzymes with enhanced targeting properties (Maga et al, 2012; Tiels et al, 2012; Zhu et al, 2009), pharmacological chaperones that assist the folding of the mutant enzyme (Okumiya et al, 2007; Parenti et al, 2007) or increase the stability of the recombinant enzyme (Porto et al, 2009, 2012), and gene therapy (Byrne et al, 2011).

A recently proposed novel approach to therapy for LSDs – modulation of transcription factor EB (TFEB) – circumvents the problem of inefficient enzyme delivery by exploiting the ability of lysosomes to expel their content into the extracellular space, thus providing clearance of the stored material (Medina et al, 2011). It has been shown that overexpression of TFEB, a master regulator of lysosomal biogenesis and autophagy, leads to the generation of new lysosomes and increased numbers of autophagosomes in a variety of cell types (Sardiello et al, 2009; Settembre & Ballabio, 2011; Settembre et al, 2011). Furthermore, since TFEB has been shown to promote lysosomal–autophagosomal fusion (Settembre & Ballabio, 2011; Settembre et al, 2011), this approach, unlike most current efforts, has the potential to prevent or resolve autophagic build-up.

Here, we have characterized the effect of TFEB overexpression on lysosomal and autophagic accumulation in previously described and newly developed PD models. Expression of TFEB in myotubes and muscle fibres resulted in lysosomal docking/fusion with the plasma membrane, lysosomal exocytosis and a dramatic reduction of intra-lysosomal glycogen accumulation. In addition, TFEB overexpression in PD muscle alleviated autophagic pathology by promoting the formation and removal of autophagolysosomes. Thus, modulation of TFEB activity holds promise for the development of a better therapy for this devastating disorder.

## RESULTS

### Establishment and characterization of new *in vitro* and *in vivo* PD models

**Figure 1:**
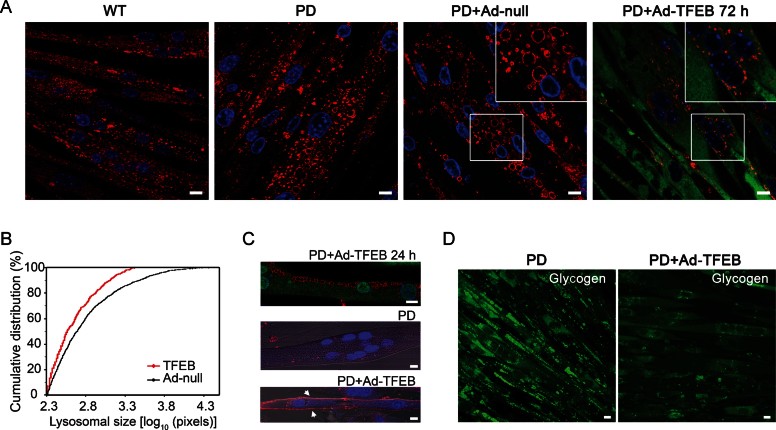
TFEB stimulates clearance of enlarged lysosomes and reduces glycogen burden in PD myotubes. A. Confocal microscopy image of PD myotubes (cl. 3LE8) infected for 72 h with adenovirus containing TFEB (PD + Ad-TFEB 72 h) shows a dramatic reduction in the number of large LAMP1-positive lysosomes (red) compared to that in untreated (PD) or adenovirus (PD + Ad-null)-treated PD myotubes. WT myotubes are shown on the left panel. Nuclei are stained with Hoechst (blue). TFEB was detected with anti-Flag antibody (green). B. Distribution of lysosomal size differs significantly in Ad-null and Ad-TFEB PD myotubes (*p* = 6.32 × 10^−8^; Kolmogorov–Smirnov test). Lysosomal size is expressed as number of pixels representing lysosomal area (LAMP1-positive structures). The median lysosomal size of Ad-TFEB infected myotubes (*m* = 367.13 pixels, *n* = 703, range 208–2659) was significantly lower than that of Ad-null infected myotubes (*m* = 491.16 pixels, *n* = 1395, range 200–2857; *p* = 6.5 × 10^−12^; Wilcoxon rank sum test). C. Confocal microscopy image of PD myotubes infected for 24 h with Ad-TFEB shows relocation of lysosomes to the plasma membrane (top). Images showing LAMP1 staining (red) on plasma membrane in a PD myotube infected with Ad-TFEB (bottom; arrows) but not in a non-infected cell (middle). Non-permeabilized cells were incubated with anti-LAMP1 antibody at 4°C for 40 min, followed by fixation and staining with secondary antibody. D. Confocal microscopy images of live non-infected PD myotubes (left) or PD myotubes infected for 72 h with Ad-TFEB (right) show a dramatic reduction in the amount of accumulated glycogen in TFEB-treated cells. The cells were incubated with the fluorescent glucose (2-NBDG; green), extensively washed, and analysed by confocal microscopy. Bar: 10 µm for all panels.

To test new therapeutic approaches for PD, we established conditionally immortalized myogenic cell lines (Supporting Information Fig S1). PD myotubes but not myoblasts or fibroblasts (Supporting Information Fig S2) replicated lysosomal pathology, namely the enlargement of lysosomes and abnormal glycogen storage (Fig 1A and D). Disappointingly, another abnormality in PD muscle fibres – autophagic accumulation (Raben et al, 2012) – was not reproduced in PD myotubes, as demonstrated by immunostaining and western analysis with LC3 [a highly specific autophagosomal marker (Kabeya et al, 2000)] (shown for western blot in Supporting Information Fig S1C).

**Figure 2:**
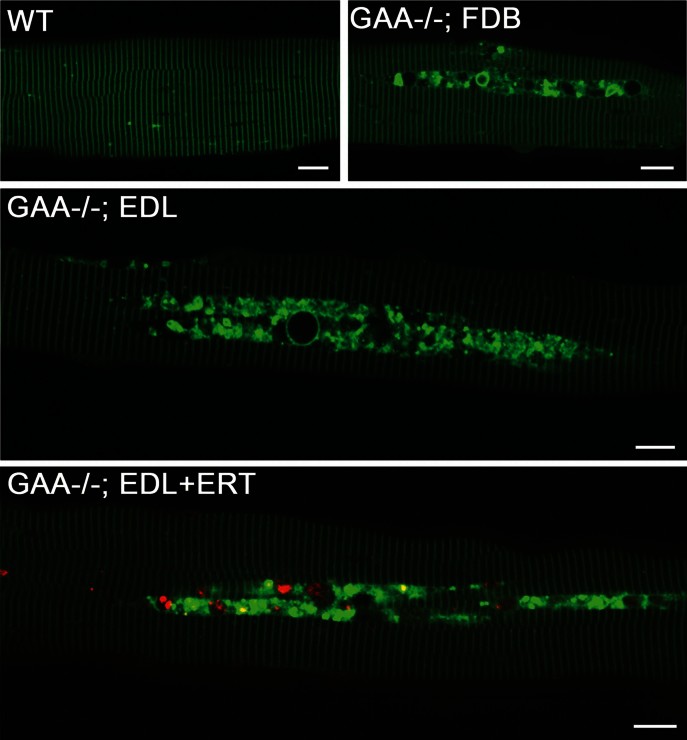
Therapeutic enzyme is trapped in the area of autophagic build-up in PD fibres. Confocal microscopy of live unstained fibres from a GFP-LC3:WT mouse (WT) and from untreated (GAA−/−) or ERT-treated (GAA−/−; +ERT) GFP-LC3:GAA−/− mice. Images show typical centrally located autophagic build-up with multiple clusters of LC3-positive autophagosomes. Structures like these are never seen in WT muscle. Autophagic build-up is present virtually in all fibres derived from EDL or gastrocnemius muscles (not shown), but only in 60–70% of FDB fibres. Labelled recombinant human GAA (red) was administered i.v. into 3–4 month-old GFP-LC3:GAA−/− mice (*n* = 5) at a dose of 100 mg/kg twice with a 24 h interval. Mice were sacrificed the next day, and live fibres (shown for EDL muscle) were analysed by confocal microscopy. The labelled recombinant GAA was detected almost exclusively in the autophagic vesicles. At least 500 fibres were analysed for each condition. Bar: 10 µm.

In contrast, autophagic pathology was clearly visible in muscle fibres from a newly developed PD mouse model, in which autophagosomes are labelled with GFP-LC3 (GFP-LC3:GAA−/−). In this new strain (but not in the myoblast cell line derived from these mice; Supporting Information Fig S3), large areas of autophagic accumulation can be seen in live myofibres without staining (Fig 2). This build-up poses an obstacle for ERT: when labelled rhGAA was administered i.v. in these mice, the drug was detected almost exclusively within autophagosomes clustered in the build-up areas (Fig 2). Thus, the culture system is useful for studying lysosomal defects, whereas the GFP-LC3:GAA−/− mouse model is suitable to address both lysosomal and autophagic abnormalities.

### TFEB overexpression reduces lysosomal size and glycogen burden in PD myotubes

To see if TFEB can promote lysosomal exocytosis and rescue lysosomal glycogen storage in multinucleated muscle cells, PD myotubes were infected with an adenovirus vector expressing Flag-TFEB (Ad-TFEB), followed by fixation and immunostaining with anti-LAMP1 (lysosomal marker) and anti-Flag antibodies.

Robust expression and nuclear staining of TFEB in myotubes were achieved after 48–72 h and resulted in a dramatic reduction of lysosomal size (*p* = 6.32 × 10^−8^; Fig 1A and B; Supporting Information Fig S4A). PD myotubes infected with the adenovirus vector (Ad-null) showed large LAMP1-positive lysosomes similar to those seen in non-infected cells (Fig 1A). Earlier, at 24 h post-infection, TFEB-expressing cells (∼10–20% of myotubes) showed a striking relocation of enlarged lysosomes toward the plasma membrane; images taken at this time point provide a snapshot of the process of lysosomal secretion (Fig 1C, top). Lysosomal exocytosis was confirmed by the release of lysosomal acid phosphatase into the culture medium (Supporting Information Table S1) and by surface LAMP1 assay, which showed the presence of lysosomal membrane marker on the plasma membrane in TFEB-treated myotubes (Fig 1C, lower) but not in untreated cells (Fig 1C, middle). TFEB also stimulated autophagy in PD myotubes, as evidenced by an increase in LC3 levels (Supporting Information Fig S4B and C).

**Figure 3:**
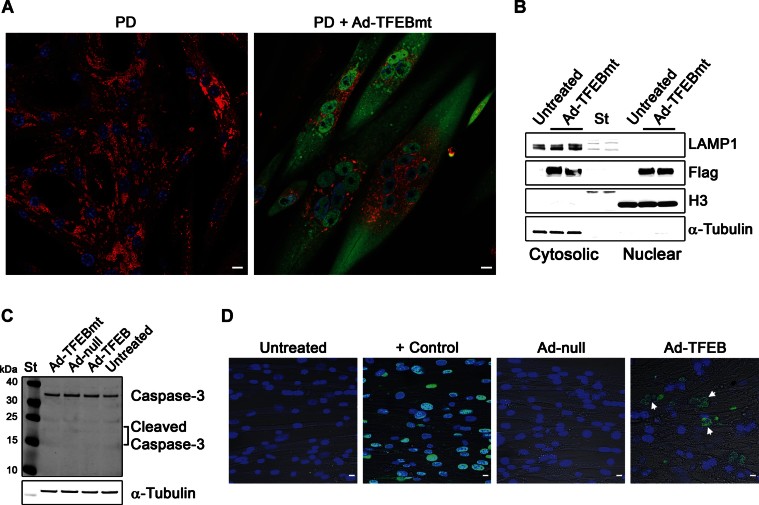
TFEBmt reduces lysosomal size, but induces apoptosis in a subset of PD myotubes. A. Immunostaining of non-infected cells (day 13 in differentiation medium) and cells infected with a mutant form of TFEB (TFEBmt) with LAMP1 (red) and Flag (green). Ad-TFEBmt was added to the myotubes for 72 h on day 10 in differentiation medium. Ad-TFEBmt-infected cells show massive accumulation of TFEB in the nuclei and significant reduction in lysosomal size, similar to that seen with TFEB. B. Western blot of cell lysates confirms the presence of TFEB in the nuclear fraction; the different intensities of the two bands corresponding to LAMP1 protein in untreated and TFEBmt-treated samples may reflect the differences in the glycosylation pattern. C. Western blot of protein lysates from untreated, Ad-null, TFEB, and TFEBmt-treated myotubes with anti-caspase-3 antibody. No activated (cleaved) products are detected in any condition. α-Tubulin was used as a loading control. D. TUNEL assay shows the presence of some apoptotic cells in Ad-TFEB-infected cultures, but not in control cultures infected with adenovirus alone (*n* = 3). Bar: 10 µm for all panels.

In addition, we tested the effect of constitutively active mutant TFEB (S211A; TFEBmt) (Martina et al, 2012; Roczniak-Ferguson et al, 2012; Settembre et al, 2012) in PD myotubes. Massive accumulation of TFEB in the nuclei resulted in a striking clearance of large lysosomes without any decrease in the total amount of LAMP1 protein (Fig 3A and B). In fact, levels of LAMP1 appear to increase in TFEBmt-treated cells, consistent with the role of TFEB in stimulating lysosomal biogenesis (Sardiello et al, 2009; Settembre & Ballabio, 2011).

As expected, the removal of enlarged lysosomes from PD myotubes was associated with a significant decrease in the amount of accumulated storage material, as shown by the incorporation of the fluorescent glucose derivative 2-NBDG into glycogen (Fig 1D). Thus, muscle cells in PD can be cleared of lysosomal glycogen accumulation by TFEB induction. Of note, many TFEB-overexpressing muscle cells, particularly those treated with TFEBmt, change their morphology: normally elongated myotubes become spindle-like and contain centrally located nuclei (Fig 3A). However, no cytotoxicity was observed in TFEB-treated cells as evidenced by lactate dehydrogenase (LDH)-measurements in culture medium (not shown). Although TUNEL assay revealed occassional apoptotic cells, no activation of caspase-3 was detected by western (Fig 3C and D).

Next, we attempted to activate endogenous TFEB by pharmacologically targeting mTORC1, which has been shown to negatively regulate TFEB (Martina et al, 2012; Roczniak-Ferguson et al, 2012; Settembre et al, 2012). Addition of Torin 1 and Torin 2 inhibited mTORC1 (Liu et al, 2010) and stimulated autophagy, but failed to reduce lysosomal size (Supporting Information Fig S5A–C; shown for Torin 2). These data suggest that the amount of endogenous TFEB may not be sufficient to support lysosomal clearance; alternatively, another kinase – MAPK (ERK1/2) kinase – may be involved in the process of TFEB activation (Settembre et al, 2011). A striking increase in the levels of p-ERK1/2 in TFEB-treated PD myotubes points to such a possibility in muscle cells (Supporting Information Fig S5D).

### TFEB overexpression in PD muscle induces cellular clearance by promoting lysosomal/autophagosomal exocytosis

To evaluate the effect of TFEB on lysosomal and autophagic pathologies *in vivo*, three knockout mouse strains were used: the previously described GAA−/− and autophagy-deficient GAA−/− (Atg7:GAA DKO) models (Raben et al, 1998, 2010), and a newly developed GFP-LC3:GAA−/− strain. Flexor digitorum brevis (FDB) muscles from each line were transfected by electroporation with plasmids containing TFEB and/or LAMP1 (Table 1). Four to six days after transfection, live single muscle fibres were analysed by time-lapse confocal microscopy; alternatively, live or fixed fibres were stained and imaged. The efficiency of the transfection in all conditions ranged from 10 to 30%.

**Table 1:** Experimental design^a^

Condition	Mouse strain	Plasmids	Rationale
1 (*n*^b^ = 73)	GFP-LC3:GAA−/−	mCherry-LAMP1	To address lysosomal–autophagosomal interactions; control for conditions 2 & 3
2 (*n* = 63)	GFP-LC3:GAA−/−	Flag-TFEB/mCherry-LAMP1	To investigate the effect of TFEB on lysosomal-autophagosomal clearance
3 (*n* = 45)	GAA−/−	GFP-TFEB/mCherry-LAMP1	To study TFEB's effect on lysosomal clearance; control for condition 4
4 (*n* = 55)	Atg7:GAA DKO	GFP-TFEB/mCherry-LAMP1	To study the effect of autophagy on TFEB-induced lysosomal clearance
5 (*n* = 29)	Atg7:GAA DKO	mCherry-LAMP1	Control for condition 4
6 (*n* = 5)	GFP-LC3:WT	mCherry-LAMP1	WT control
7 (*n* = 47)	GAA−/−	GFP-TFEB	To label mitochondria and lysosomes in live and fixed fibres

a 3–4 animals were used for conditions 1–6; 2 animals were used for condition 7. For each animal ∼1000 fibres were isolated.

b *n* = number of fibres analysed by confocal microscopy.

**Figure 4:**
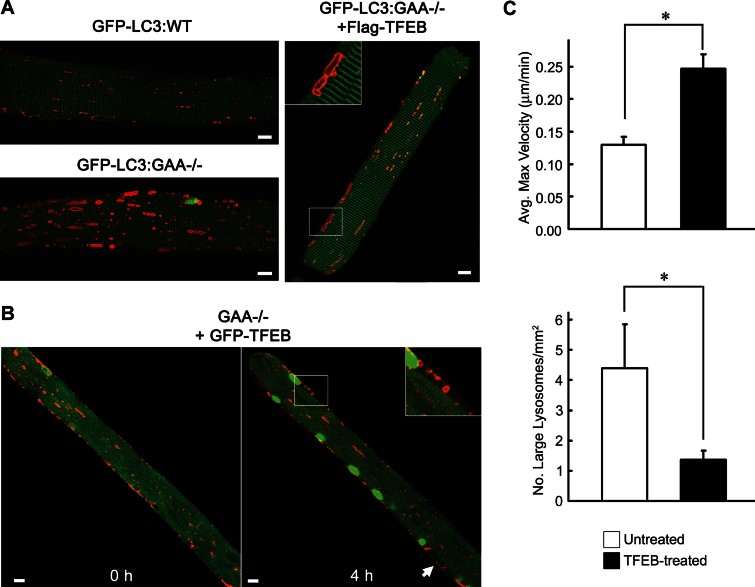
TFEB promotes clearance of enlarged lysosomes and increases lysosomal motility in PD fibres. A. Confocal microscopy images of live fibres derived from 3 to 4 month-old GFP-LC3:WT (top left), untreated GFP-LC3:GAA−/− (bottom left) or TFEB-treated GFP-LC3:GAA−/− (right) mice. All fibres were transfected with mCherry-LAMP1 to visualize lysosomes (red). The effects of TFEB are clearly visible – overall reduction in lysosomal size, appearance of normal size lysosomes (similar to those in the WT), and lysosomal docking to the plasma membrane (inset). Bar: 10 µm. B. FDB muscle of a GAA−/− mouse was transfected with both GFP-TFEB and mCherry-LAMP1 (GAA−/− + GFP-TFEB). Images were taken before (left) and after 4 h (right) of time-lapse microscopy. Lysosomal clearance is visible in the TFEB-transfected fibre at both time points. Lysosomes appear to “exit” the fibre (inset and arrow) at the 4 h time point when TFEB is activated as evidenced by its nuclear translocation (green nuclei). Bar: 10 µm. C. The mean maximum velocity of lysosomes (top) and the number of large (>3.5 µm) lysosomes (bottom) in untreated and TFEB-treated PD fibres (note: all data for Flag- and GFP-TFEB-treated fibres are pooled). Lysosomal velocities were calculated from time-lapse images using ImageJ software with the manual tracking plug-in. For each condition the trajectories of multiple lysosomes were followed (*n* = 26 untreated; *n* = 43 TFEB-treated) and the three highest velocity measurements per lysosome were recorded. In TFEB-treated fibres, the maximum velocity of lysosomes was significantly increased (*p* = 2.07 × 10^−17^) and the number of large lysosomes was significantly decreased (*p* = 1.0 × 10^−3^). Ten untreated and 24 TFEB-treated fibres were analysed for the size calculations. * indicates statistically significant differences (*p* ≤ 0.001; Student's *t*-test). Error bars represent 95% confidence intervals.

Both Flag-TFEB/mCherry-LAMP1- and GFP-TFEB/mCherry-LAMP1-transfected PD fibres (Table 1, conditions 2 and 3) showed a striking decrease in the number of large lysosomes, translocation of enlarged lysosomes to the periphery of the fibre, docking and fusion of lysosomes with the plasma membrane, the emergence of multiple normal dot-like size lysosomes, and a markedly increased motility and fusion of lysosomes (Fig 4A–C; Movie 1) compared to untreated PD controls (Table 1, condition 1; Movie 2). The mean maximum velocity of lysosomes in TFEB-treated fibres was 0.247 ± 0.01 µm/min [pooled data from conditions 2 (*n* = 24) and 3 (*n* = 19)], whereas this value was 0.130 ± 0.006 µm/min (*n* = 26) in untreated controls (*p* = 2.07 × 10^−17^). Importantly, the number of large (>3.5 µm in length) lysosomes was significantly reduced in TFEB-treated fibres [4.41 ± 0.7 and 1.37 ± 0.2 lysosomes/mm^2^ for untreated (*n* = 10) and treated (*n* = 24) fibres, respectively; *p* = 1.0 × 10^−3^] (Fig 4C; Supporting Information Fig S6A). This dramatic decrease in the number of large lysosomes was confirmed by lysotracker and LAMP1 staining of live or fixed fibres transfected with GFP-TFEB alone (condition 7; Supporting Information Fig S6B and C).

The vast majority of TFEB-transfected fibres (freshly isolated or maintained for days in culture) had normal shape and appearance, suggesting that overexpression of TFEB does not cause any appreciable muscle damage. Furthermore, no major mitochondrial abnormalities were observed in TFEB-treated fibres as evidenced by MitoTracker labelling and immunostaining for cytochrome c (Supporting Information Fig S7A and B). However, stress of fibres exposed to hours of time-lapse microscopy resulted in a massive translocation of TFEB into the nuclei and – in occasional fibres – the detachment of plasma membrane (Fig 4B, arrow and inset; Supporting Information Fig S8A; Movie 3). This phenomenon was not seen in control experiments, in which the muscle of GFP-LC3:GAA−/− mice was transfected with mCherry-LAMP1 only (Table 1, condition 1).

The choice of the GFP-LC3:GAA−/− strain for control experiments enabled us not only to evaluate the motility of lysosomes, but also to examine the possibility of impaired lysosomal/autophagosomal fusion as a mechanism underlying autophagic build-up. Unexpectedly, the expression of mCherry-LAMP1 was seen almost exclusively in fibres or in regions of fibres free from autophagic build-up (∼80–90% of all transfected fibres exhibited these two expression patterns in roughly equal proportions; Supporting Information Fig S8B), suggesting a compromised lysosomal biogenesis in areas of autophagic accumulation. However, in those fibres in which mCherry-LAMP1 was expressed in the build-up area (∼10–20%), lysosomal-autophagosomal fusion events appear rare or non-existent (Movie 4).

It was reasonable to assume that TFEB overexpression may increase lysosomal–autophagosomal fusion, thus preventing or even resolving autophagic build-up in PD muscle. Indeed, none of the Flag-TFEB/mCherry-LAMP1 transfected fibres from GFP-LC3:GAA−/− mice (Table 1, condition 2) had well-defined autophagic areas, suggesting autophagosomal clearance. We did see occasional fibres (<1%; 5/800 fibres) with clusters of autophagosomes reminiscent of the build-up. The lengths of these clusters, however, did not reach the size of a typical build-up, which commonly spans more than 100 µm, often extending the whole length of the fibre (with or without interruptions) in untreated PD muscle.

**Figure 5:**
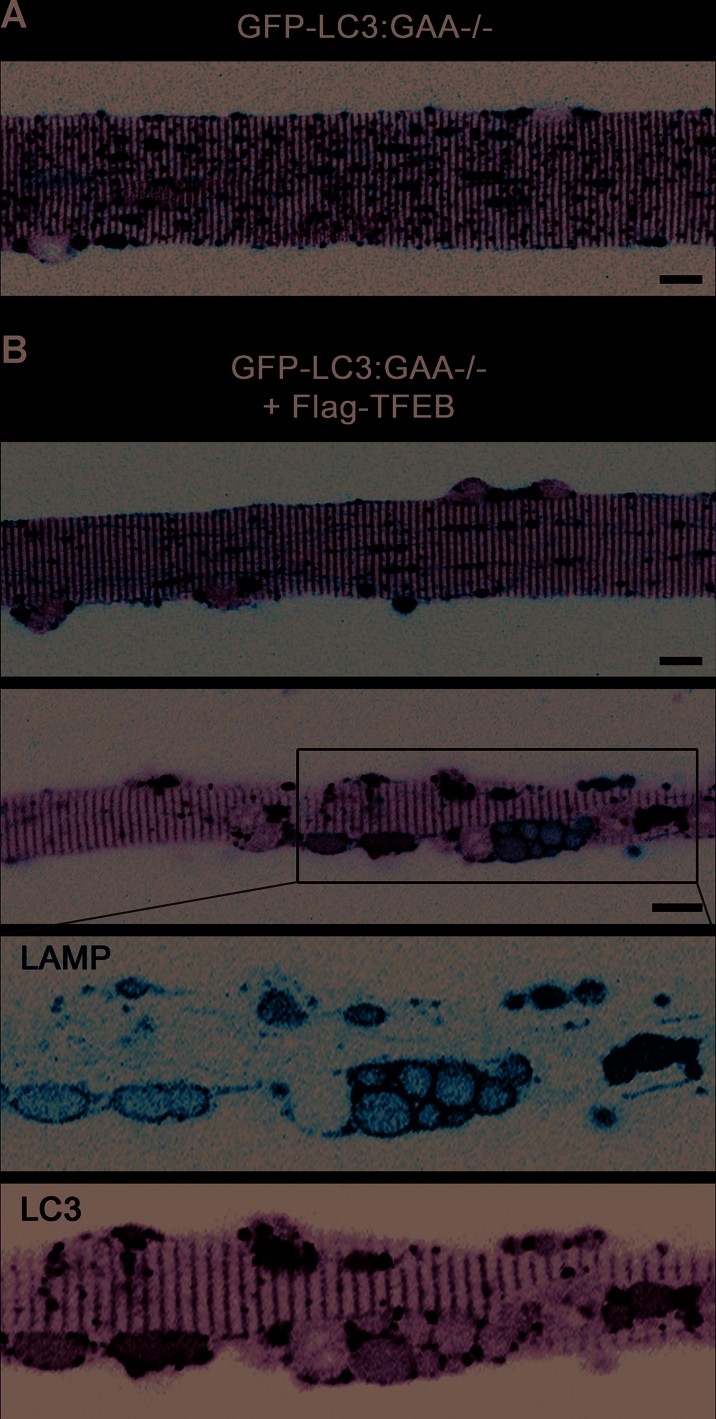
TFEB stimulates lysosomal–autophagosomal fusion and clearance. Confocal microscopy images of live fibres from GFP-LC3:GAA−/− mice. Muscle was transfected with mCherry-LAMP1 only. The image (a single frame from the time-lapse series presented in Movie 5) shows lysosomes (red), LC3-positive autophagosomes (green) and a number of autolysosomes (yellow). Muscle was transfected with Flag-TFEB and mCherry-LAMP1. Massive formation of autolysosomes is indicated by yellow structures; the three lower panels provide a snapshot of the process of exocytosis. The structures at the plasma membrane are labelled with both LC3 and LAMP1, indicating that they represent amphisomes (a product of fusion between autophagic vesicles and late endosomes) or autolysosomes (a product of fusion between autophagic vesicles and lysosomes). Bar: 10 µm for all panels.

To analyse the effect of TFEB on lysosomal–autophagosomal fusion in the absence of clear-cut autophagic build-up, we took advantage of stress-induced surges in the number of LC3-positive autophagosomes during time-lapse microscopy. During these surges, TFEB-treated fibres (Table 1, condition 2) exhibited a striking increase in lysosomal–autophagosomal fusion compared to build-up free control fibres (transfected with mCherry-LAMP1 only; condition 1; Movies 5 and 2), as indicated by co-localization of LAMP1 (red) and LC3 (green) markers (Fig 5; Movie 6). Furthermore, the transfected fibres showed alignment of doubly labelled autophagolysosomes (rather than vesicles labelled by LAMP1 only) along the plasma membrane (Fig 5B, three lower panels; Movie 7), suggesting that TFEB may induce lysosomal/autophagosomal exocytosis. These data raised an intriguing possibility that autophagy may facilitate TFEB-induced lysosomal clearance.

### Suppression of autophagy attenuates TFEB-mediated cellular clearance in PD muscle

**Figure 6:**
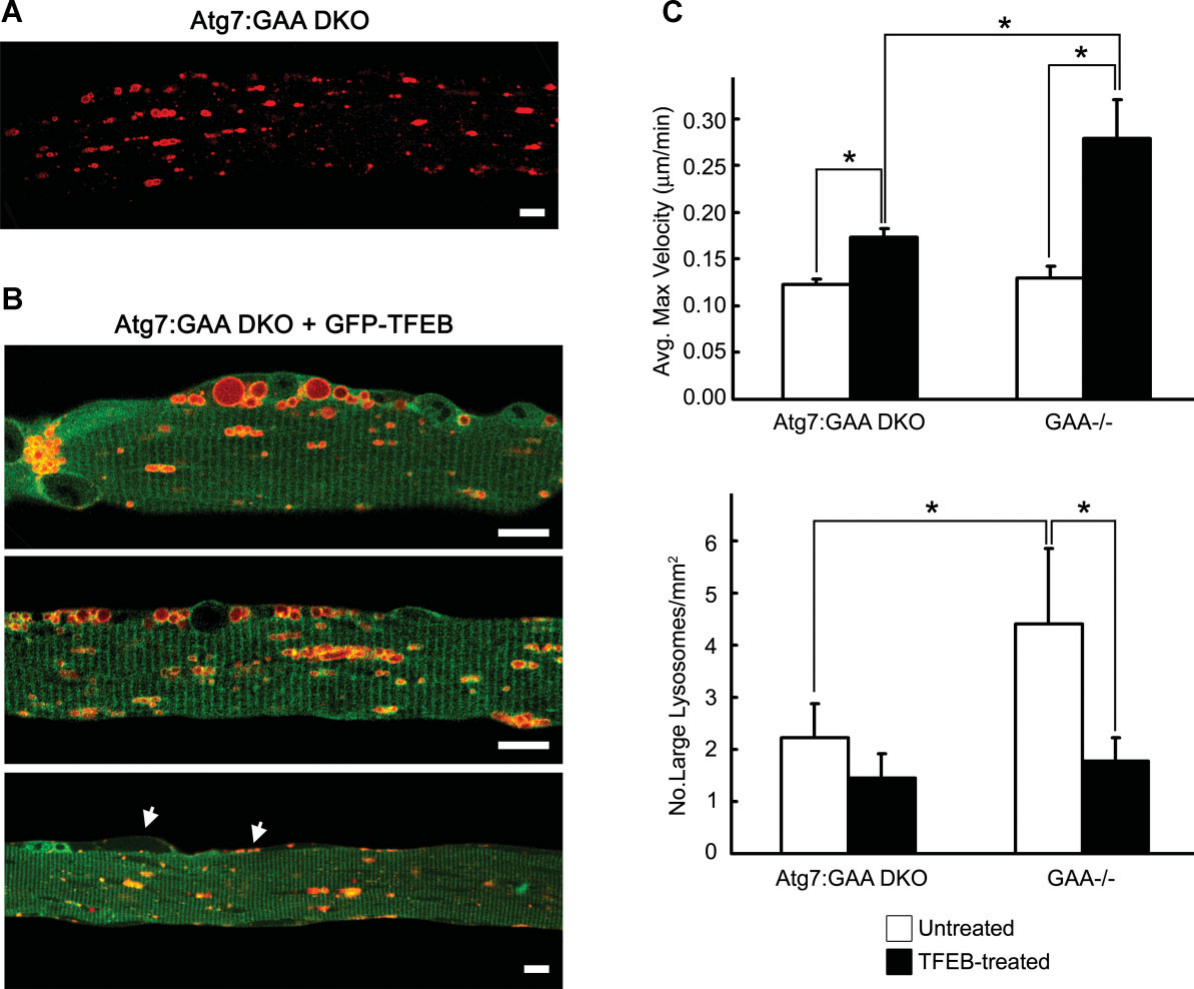
TFEB promotes redistribution and docking of lysosomes to the plasma membrane in autophagy-deficient PD fibres. Confocal microscopy images of live fibres from muscle-specific autophagy-deficient GAA−/− mice (Atg7:GAA DKO). Muscle was transfected with mCherry-LAMP1 only. Muscle was transfected with GFP-TFEB and mCherry-LAMP1 (Atg7:GAA DKO +GFP-TFEB). The TFEB-transfected fibres show realignment of the lysosomes and membrane detachment (most striking in top and bottom panels) similar to those in TFEB-transfected fibres from PD mice (see Fig 4B inset). Lysosomes can be seen in the space between the fibre and plasma membrane (arrows). Bar: 10 µm for all panels. The mean maximum velocity of lysosomes (top) and the number of large (>3.5 µm) lysosomes (bottom) in untreated and TFEB-treated autophagy-deficient Atg7:GAA DKO fibres. The increase in maximum velocity (41%) is significant (*p* = 2.729 × 10^−18^; *n* = 57 lysosomes for untreated; *n* = 52 lysosomes for TFEB-treated), but there is only a slight trend toward smaller lysosomal size (*p* = 7.0 × 10^−2^; *n* = 10 fibres for untreated; *n* = 21 fibres for TFEB-treated). The corresponding values from GAA−/− mice are presented for comparison (for TFEB-treated condition: *n* = 19 lysosomes for velocity measurements, and *n* = 10 fibres for the lysosomal size measurements). * indicates statistically significant differences (*p* ≤ 1.0 × 10^−5^ for the top panel and *p* ≤ 0.01 for the lower panel; Student's *t*-test).

To address the role of autophagy, GFP-TFEB and mCherry-LAMP1 were introduced into muscle-specific autophagy-deficient GAA−/− mice (Atg7:GAA DKO; Table 1, condition 4). The telltale signs of TFEB's effect on lysosomal pathology – redistribution and docking along the plasma membrane, and even membrane blebbing – were evident in autophagy-deficient PD muscle fibres (Fig 6B). However, the strikingly active lysosomal movement and fusion seen in TFEB-treated PD fibres (Table 1, condition 3; Movie 1) were not observed when autophagy was suppressed. The maximum velocity of lysosomes (0.174 ± 0.005 µm/min; *n* = 52) was significantly lower than that in TFEB-treated PD mice (0.279 ± 0.02; *p* = 7.01 × 10^−6^; *n* = 19) (Fig 6C, top; Movie 8). Furthermore, the TFEB-induced increase in lysosomal velocity seen in fibres with genetically intact autophagy (115%) was greatly attenuated in autophagy-deficient PD fibres (a 41% increase). This modest effect of TFEB was associated with inefficient cellular clearance as evidenced by only a slight decrease in the number of large (>3.5 µm in length) lysosomes compared to controls (condition 5; Fig 6C, bottom). This limited effect is particularly striking given the already decreased baseline lysosomal size (*p* = 9.6 × 10^−3^; Fig 6A and C) and a previously reported reduction of glycogen levels in autophagy-deficient PD muscle (Raben et al, 2010). Altogether, the data suggest that autophagy is a prerequisite for efficient TFEB-mediated cellular clearance.

### Intramuscular injection of AAV2.1-TFEB results in clearance of glycogen stores and amelioration of muscle pathology

To test the effects of TFEB overexpression on muscle pathology in PD mice, 1-month-old GAA−/− mice received direct intramuscular injections of an AAV2.1-TFEB vector in three sites of the right gastrocnemius muscle. AAV2.1-GFP vector or vehicle alone was injected into the contralateral muscle. The animals were sacrificed 45 days after the injection to allow maximal, sustained expression of the vector. Levels of TFEB expression, analysed by real-time PCR, were 10-fold higher in the AAV2.1-TFEB-injected muscles compared to those in controls.

**Figure 7:**
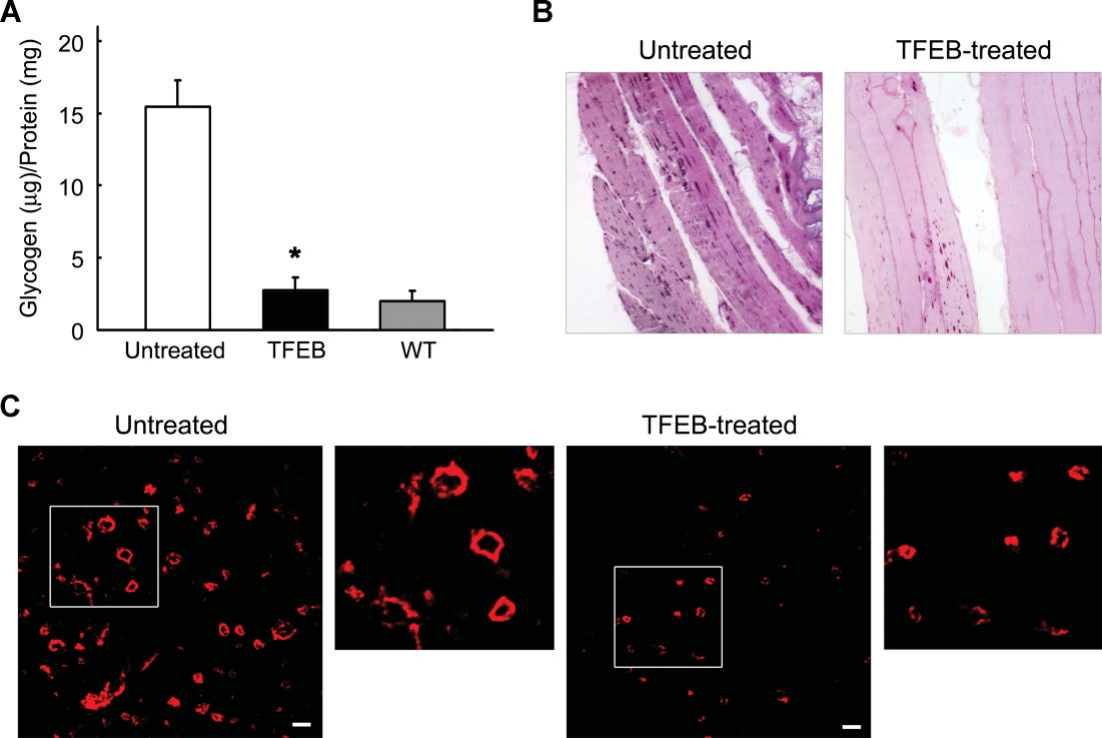
Intramuscular injection of AAV2.1-TFEB in GAA −/− mice promotes glycogen clearance and attenuates PD pathology. A. Glycogen assay in TFEB-injected gastrocnemii and in the contralateral muscles. In TFEB-injected muscles glycogen levels were significantly decreased compared to those in untreated muscles. * indicates statistically significant differences (*p* = 1.0 × 10^−4^; *n* = 6; Student's *t*-test). B. PAS staining of TFEB-treated muscle shows a reduction of lysosomal glycogen stores (puncta) compared to those in untreated muscle. Original magnification: 20×. C. LAMP1 staining of TFEB-injected gastrocnemii and of the contralateral untreated muscles. In TFEB-treated muscles, the size of LAMP1-positive vesicles was reduced. Bar: 2 µm.

TFEB expression resulted in near-complete clearance of accumulated glycogen (2.8 ± 0.88 µg glycogen/mg protein as opposed to 15.48 ± 1.80 in untreated muscle, *p* = 1.0 × 10^−4^; glycogen levels in WT muscle were 2.01 ± 0.70) (Fig 7A). The reduction of lysosomal glycogen stores (glycogenosomes) in TFEB-treated muscle was confirmed by PAS staining and by a decrease in the size of LAMP1-positive vesicles (Fig 7B and C). Haematoxylin–eosin staining did not show any signs of toxicity or gross alterations of the muscle architecture in TFEB-treated muscle (Supporting Information, Fig S9A). Compared to controls, there was no increase in the number of apoptotic cells or signs of caspase-3 activation (Supporting Information, Fig S9B and C).

No significant differences in muscle size were observed between untreated and TFEB-treated muscles (Supporting Information Fig S9D and E).

**Figure 8:**
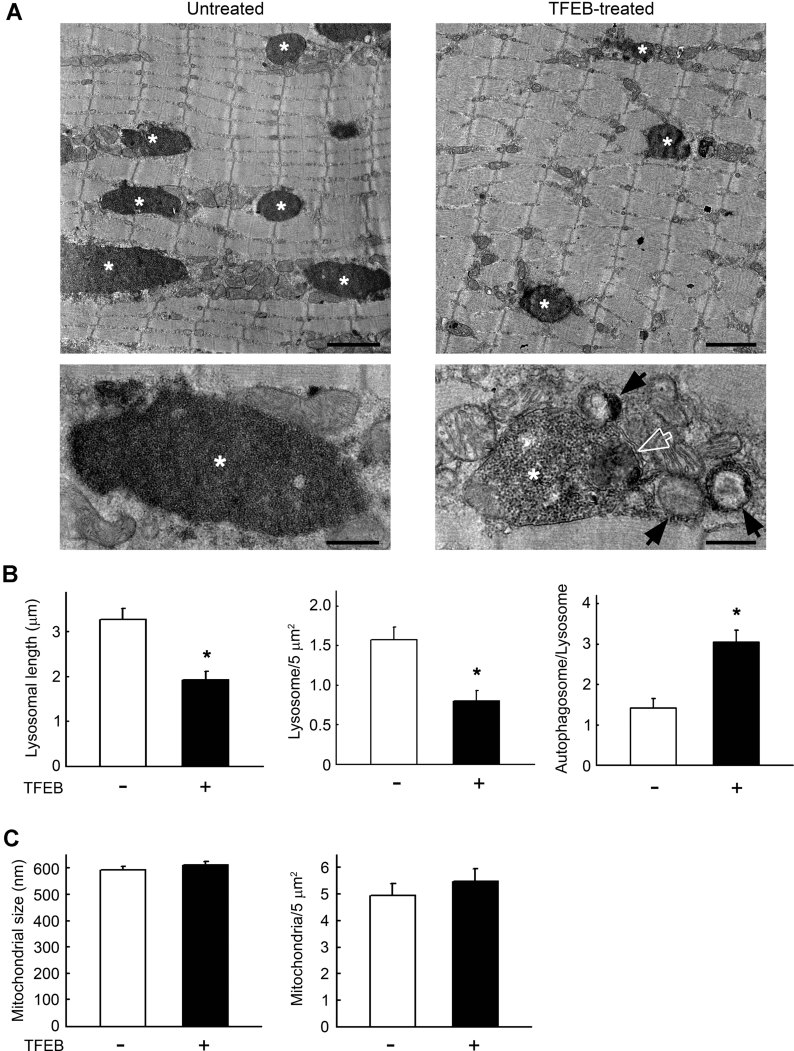
Impact of TFEB on muscle fibre ultrastructure in GAA−/− mice. A. EM images of muscle injected with either AAV-GFP (untreated) or AAV-TFEB (TFEB-treated). Asterisks indicate glycogen-containing lysosomes. Bar: 1.5 µm (upper panels) and 0.45 µm (lower panels). Higher magnification images (lower panels) show that glycogen particles are less densely packed in TFEB-treated muscle. Black arrows indicate autophagosome profiles; the white empty arrow shows remnants of mitochondria engulfed by the lysosome. B. Graphical presentations of lysosomal length (average ± SE; *n* = 100 lysosomes; *p* = 6.31 × 10^−5^), the number of lysosomes per 5 µm^2^ area of muscle fibre section (average ± SE; *n* = 50 fields; *p* = 4.80 × 10^−3^), and the number of autophagosomes flanking glycogen-containing lysosomes (average ± SE; *n* = 100 lysosomes; *p* = 4.39 × 10^−5^ < 0.001). Student's *t*-test was used for each comparison. C. Graphical presentations of mitochondrial size (average ± SE; *n* = 100) and the number of mitochondria per 5 µm^2^ area of muscle fibre section (average ± SE; *n* = 50 fields). The differences were not significant by Student's *t*-test (*p* = 3.65 × 10^−1^ and 4.27 × 10^−1^, respectively).

EM analysis of TFEB-injected muscle showed a significant improvement of muscle ultrastructure – a clear reduction in the size and number of glycogen-containing lysosomes compared to those in PD muscle (Fig 8A and B left and middle panels). Furthermore, the intralysosomal electron-dense glycogen particles seen in untreated fibres (Fig 8A, bottom left, asterisk) showed looser organization in TFEB-treated muscle (Fig 8A, bottom right, asterisk). An increased number of autophagosomes in close proximity to glycogen-containing organelles was also observed in TFEB-treated muscle (Fig 8A, bottom right, black arrows; Fig 8B right panel). Notably, some autophagosomes contained glycogen particles as well, most likely derived from the cytosol. In addition, lysosomes frequently contained remnants of other intracellular organelles in their lumens (Fig 8A, bottom right, empty arrow), indicating active fusion with neighbouring autophagosomes.

The number of mitochondria in TFEB-treated fibres was comparable to that in untreated PD mice, and the size and morphology of mitochondria were normal (Fig 8C). An increased number of mitophagic profiles was also detected in treated muscles (not shown), due to the general activation of autophagy. Overall, the data on intramuscular injection indicate that TFEB is able to significantly rescue glycogen storage and morphological abnormalities.

## DISCUSSION

We have shown that TFEB efficiently triggers lysosomal exocytosis and promotes cellular clearance in muscle cells, isolated live muscle fibres, and the whole muscle of PD mice.

The process of regulated lysosomal exocytosis – which underlies TFEB-mediated cellular clearance – was initially ascribed to a subset of secretory lysosomes (or lysosome-related organelles) that reside in particular cell types, such as haematopoietic cells or melanocytes (Blott & Griffiths, 2002). However, it has been shown that conventional lysosomes can also undergo the same process of docking to the plasma membrane, followed by fusion with the membrane and a discharge of lysosomal content (Andrews, 2000). This synaptotagmin-regulated Ca^2+^-dependent process is considered to be critical for the repair of plasma membrane lesions (Rao et al, 2004).

Interestingly, lysosomal exocytosis has been implicated as a mechanism of cellular clearance in cell models of several LSDs, such as metachromatic leukodystrophy and mucolipidosis type I (Klein et al, 2005; Yogalingam et al, 2008). Furthermore, it has been shown that lysosomal exocytosis is the mechanism by which 2-hydroxypropyl-β-cyclodextrin – an approved drug for yet another LSD, Niemann Pick Type C (NPC) disease – alleviates cholesterol storage in NPC cells (Chen et al, 2010). Similarly, δ-tocopherol was shown to improve the phenotypes in several LSD cell models by stimulating Ca^2+^-mediated lysosomal exocytosis (Xu et al, 2012).

However, it was not until the discovery of TFEB that the stimulation of lysosomal exocytosis and biogenesis became a therapeutic option for LSDs and other disorders with accumulation of abnormal proteins (Medina et al, 2011; Sardiello et al, 2009; Settembre & Ballabio, 2011; Settembre et al, 2011). Overexpression of TFEB induced lysosomal exocytosis in a mouse model of multiple sulfatase deficiency (MSD) and in cells derived from the murine models of mucopolysaccharidosis type IIIA (MPS-IIIA), neuronal ceroid lipofuscinoses, and MSD (Medina et al, 2011). The effect of TFEB was also demonstrated in fibroblasts from a PD patient (Medina et al, 2011), but it remained unclear whether TFEB would have similar effects in terminally differentiated multinucleated muscle cells and PD muscle, the tissue that presents the greatest challenge for ERT.

In this study, we have demonstrated that the number of large glycogen-filled lysosomes and the amount of accumulated glycogen – the hallmarks of PD – were significantly reduced in both cultured myotubes and muscle fibres, thus providing strong evidence of TFEB potential as a therapeutic target in PD.

The simplicity of detecting hugely expanded lysosomes and glycogen in PD myotubes made this cell model particularly attractive: the effects of TFEB were easily monitored and evident within days. In TFEB-treated myotubes, the lysosomal marker LAMP1 – a protein typically present inside the cells – was seen on the outer plasma membrane. Furthermore, the release of lysosomal acid phosphatase from TFEB-treated cells provided additional evidence that lysosomal clearance proceeds through exocytosis in muscle cells, consistent with findings in other cell types (Medina et al, 2011). The cell system also allowed us to expose a potential problem with high TFEB activity; a constitutively active form of TFEB, in which S211 is mutated to alanine (Martina et al, 2012), invariably resulted in morphological changes of myotubes, perhaps because of massive expulsion of the lysosomes.

Live fibre experiments employing time-lapse confocal microscopy provided a unique opportunity to track lysosomal movements and fusion, to estimate the motility of lysosomes, and to examine the interactions between lysosomes and autophagosomes following TFEB activation and nuclear translocation. Similar to what was observed in the cell system, *in vivo* TFEB delivery efficiently triggered lysosomal exocytosis and promoted cellular clearance, as evidenced by a significant reduction in glycogen levels and the number of large lysosomes in PD muscle. Thus, lysosomal pathology in PD muscle can be corrected by TFEB overexpression.

The development of a new PD mouse model, the GFP-LC3:GAA−/− strain, allowed us to address the effect of TFEB on autophagic accumulation – the major secondary abnormality in PD skeletal muscle (Raben et al, 2007a, b, 2012). Defects in autophagy, a major lysosome-dependent degradative system (Yang & Klionsky, 2010)], significantly contribute to the pathophysiology of several lysosomal storage diseases (Cao et al, 2006; Cox & Cachon-Gonzalez, 2012; Fukuda et al, 2006a, b; Liao et al, 2007; Lieberman et al, 2012; Settembre et al, 2008). In PD skeletal muscle, the autophagic defect is particularly striking, manifesting as a massive build-up which poses an additional problem for ERT.

Remarkably, none of the TFEB-transfected fibres had large areas of autophagic build-up, which are so prominent in most non-transfected PD fibres. The lack of typical build-up suggests that TFEB may have rescued autophagic pathology in PD muscle. The mechanism of this rescue is not clear, but the unexpected surge in autophagy – a dramatic increase in the number of LC3-positive autophagosomes during hours of time-lapse microscopy – provided a clue. TFEB-treated fibres exhibited a significant increase in fusion between LC3- and LAMP1-positive vesicles, an appearance of multiple LC3/LAMP1-positive autophagolysosomes, and docking of these double-positive structures to the plasma membrane. These data suggest that TFEB may relieve autophagic build-up by stimulating the formation and secretion of autophagolysosomes – a product of lysosomal-autophagosomal fusion.

We hypothesize that lysosomal exocytosis may in fact not be a purely lysosomal event, but rather a process involving autophagy and secretion of autophagolysosomes. Our data on the much attenuated effects of TFEB on lysosomal velocity and clearance in autophagy-deficient PD mice support this idea. Lysosomal function depends on the ability of lysosomal membranes to fuse with other vesicles' membranes and with the plasma membrane (Luzio et al, 2007). Abnormal accumulation of cholesterol in the endolysosomal membranes in cell models of two neurodegenerative LSDs (MSD and MPS-IIIA) was shown to sequester SNARE receptors – essential components of the membrane fusion machinery – in cholesterol-enriched membrane regions, resulting in a significantly reduced ability of lysosomes to fuse with vesicles of endocytic and autophagic pathways (Fraldi et al, 2010). Perhaps the merging of lysosomal and autophagosomal membranes is required for successful fusion with the plasma membrane and for fully efficient TFEB-mediated cellular clearance.

Two kinases, mTORC1 and ERK, have been implicated in TFEB regulation in different cells (Martina et al, 2012; Roczniak-Ferguson et al, 2012; Settembre et al, 2011, 2012). The regulation of TFEB in skeletal muscle remains an open question, which is beyond the scope of this paper. An unexpected activation of ERK in TFEB-overexpressing cells could be an attempt to down-regulate TFEB and restore homeostasis; it is also possible that activation of ERK could be a downstream event. Whatever the case, the search for compounds which can activate TFEB in muscle would benefit the development of a TFEB-mediated therapeutic approach. In thinking about TFEB as a target for potential therapeutic intervention, one can speculate that a single event of TFEB-mediated clearance of the stored substrate and autophagic material may provide prolonged relief since the rate of muscle glycogen accumulation in late-onset PD is relatively slow. Furthermore, ERT may function more efficiently in TFEB-treated muscle, particularly because of the reduction in autophagic build-up. A combination of TFEB-targeted therapy with ERT, perhaps at much lower dosages than those currently employed (and therefore at reduced cost), could then maintain muscle tissue in normal or nearly normal condition. In the meantime, pre-clinical studies are needed to fully evaluate the consequences of TFEB overexpression on muscle function and to exclude long-term toxicity.

Finally, the potential applications of our newly developed models, PD myotubes and GFP-LC3:GAA−/− mice, extend beyond testing the effects of TFEB. For example, an *in vitro* PD model is much needed for large-scale drug testing. Recently, several attempts have been made to develop such a model: these include the generation of cardiomyocyte-like cells from human induced pluripotent stem (iPS) cells (Huang et al, 2011), muscle cells from reprogrammed murine iPS cells (Kawagoe et al, 2011), and murine muscle cells transduced by viral constructs containing immortalizing genes (Douillard-Guilloux et al, 2009; Takikita et al, 2009). Our new PD muscle cell lines have several advantages: they are highly myogenic, derived from individual clones and, most importantly, they maintain the capacity to differentiate when the immortalizing gene, shown to perturb myogenic program (Douillard-Guilloux et al, 2009; Mouly et al, 1996), is inactivated. These cell lines replicate lysosomal pathology but fail to recapitulate autophagic build-up. Possible explanations for this failure – common to all *in vitro* PD models – are the relatively short survival time of myotubes in culture and the need for nutrient-rich conditions (Fig S3C). Additional studies are needed to find a way to “trick” these cells into displaying the autophagic accumulation that is so striking in Pompe muscle fibres.

In the interim, the newly generated GFP-LC3:GAA−/− mouse model provides an excellent tool to monitor autophagic accumulation in live fibres, in muscle bundles, and even in muscle of a living mouse [*i.e.* by intravital imaging (our unpublished data)]. In addition to using these mice to address TFEB's effects, we have utilized the model to examine intracellular trafficking of rhGAA and to explore possible mechanisms of autophagic build-up. The near absence of lysosomal–autophagosomal fusion and strikingly low expression of LAMP1 in the build-up area suggest inefficient local lysosomal biogenesis. LAMPs are considered to be of key importance for lysosomal movement, maturation of autophagosomes and lysosomal exocytosis (Saftig & Klumperman, 2009). Therefore, these low local LAMP1 concentrations in the build-up area may have far reaching consequences. The build-up process appears to begin with the failure of a subset of lysosomes to fuse with autophagosomes, perhaps because of lysosomal rupture during muscle contractions. It is possible that this fusion defect and the paucity of the newly formed lysosomes create conditions that favour expansion of the autophagic build-up. With the capacity to resolve both of these deficits by promoting lysosomal–autophagosomal fusion and biogenesis, TFEB is uniquely suited to remedy autophagic and lysosomal pathologies in PD.

## MATERIALS AND METHODS

Antibodies: rat anti-LAMP1 (1DB4), mouse anti-cytochrome c, and mouse anti-MyoD (MoAb 5.8A) (BD Pharmingen, San Diego, CA); rabbit anti-LC3B and mouse anti-Flag (M2) (Sigma–Aldrich, St. Louis, MO); mouse anti-myogenin (F5D) (Dako, Glostrup, Denmark); mouse anti-myosin heavy chain (Clontech, Mountain View, CA); rabbit anti-caspase-3, rabbit anti-p44/42 MAPK (Erk1/2), rabbit anti-phospho-p44/42 MAPK (Erk1/2; Thr202/Thr204), rabbit anti-phospho-4EBP1 (Thr37/46), and rabbit anti-Histone H3 (D1H2) (Cell Signaling, Beverly, MA). Rabbit anti-alpha tubulin (Abcam, Cambridge, MA) or mouse anti-vinculin antibody (Sigma–Aldrich) was used as a loading control for western blot. Alexa Fluor-conjugated secondary antibodies (Life Technologies, Grand Island, NY) were used for immunostaining and western blot analysis. Alexa Fluor 546 labelled recombinant human GAA (6.2 mg/ml) was provided by Genzyme Corp. (Framingham, MA).

Plasmids: plasmid containing the full-length 3xFlag-tagged human TFEB (referred to as Flag-TFEB) was described previously (Sardiello et al, 2009); plasmid containing the full-length rat LAMP1 (mCherry-LAMP1) was obtained from Dr. Kristien Zaal (Light Imaging Section, Office of Science & Technology, NIAMS, NIH, Bethesda, MD, USA); plasmid-containing GFP-TFEB was described previously (Martina et al, 2012).

### Generation of mice and isolation of single live myofibres

The generation of GFP-LC3:GAA−/−, immorto:GAA−/−, and immorto:GFP-LC3:GAA−/− mice is described in the Supporting Information. Single live myofibres from gastrocnemius and extensor digitorum longus (EDL) muscles from 3 to 4 month-old mice were isolated as described (Rosenblatt et al, 1995). Isolation of single live fibres from FDB muscle was done with some modifications of the previously published protocol (Raben et al, 2010) (Supporting Information). Live fibres were either monitored by time-lapse microscopy or used for labelling acidic organelles [LysoTracker® Red DND-99 (Life Technologies), 100 nM] or mitochondria [MitoTracker® Red CMXRos (Life Technologies), 100 nM]. Alternatively, fibres were fixed in 2% paraformaldehyde for 30 min and immunostained with anti-cytochrome c and anti-LAMP1 antibodies. Both live and fixed fibres were analysed on a LSM 510 confocal microscope (Carl Zeiss, Göttingen, Germany; see Supporting Information) equipped with Plan-Neofluar 40X oil immersion objective.

**The paper explained**

**PROBLEM**

The major problem with the current ERT for several LSDs is inefficient drug delivery to lysosomes in particular target tissues. Furthermore, the therapy does not address autophagic abnormalities, which are commonly found in LSDs. These limitations are apparent in PD, a severe metabolic myopathy due to a deficiency of glycogen-degrading lysosomal enzyme, GAA. Experimental data and clinical experience over the past decade provide strong evidence of skeletal muscle resistance to therapy with recombinant human GAA. Here, we have tested a recently developed approach for therapy of LSDs in PD mouse models. This new approach circumvents the problem of ineffective drug delivery and involves manipulation of TFEB.

**RESULTS**

For testing the novel therapeutic approach, we developed new PD models. Experiments in PD cell culture and animal models demonstrate that TFEB has the capacity to rid muscle cells of excessive glycogen burden and accumulation of autophagic debris. Overexpression of TFEB in muscle cells or in Pompe mice stimulated fusion between lysosomes and autophagosomes, resulting in an increased formation and exocytosis of autolysosomes. Unexpectedly, the effects of TFEB were almost abolished when autophagy was genetically suppressed.

**IMPACT**

Our study provides strong evidence that TFEB is a valid therapeutic target for PD as well as other LSDs. The appeal for PD is that unlike the current therapy, modulation of TFEB holds promise to address both lysosomal and autophagic pathologies in skeletal muscle. The study also reveals a previously unrecognized role of autophagy in TFEB-mediated cellular clearance.

### *In vivo* electroporation of skeletal muscle and enzyme replacement therapy

Electric pulse-mediated gene transfer into FDB muscle was performed as described (DiFranco et al, 2009). Three to four month-old GAA−/−, GFP-LC3:GAA−/−, or muscle-specific autophagy-deficient GAA−/− (MLCcre:Atg7F/F:GAA−/−; referred to as Atg7:GAA DKO) mice were used for the experiments (Table 1). The animals were sacrificed 4–6 days after the procedure. Live FDB fibres were isolated and analysed by time-lapse confocal microscopy. Alexa-Fluor 546 labelled recombinant human GAA (rhGAA) was administered into 3–4 month-old GFP-LC3:GAA−/− mice at a dosage of 100 mg/kg twice with a 24 h interval. Mice were sacrificed 24 h after the last injections. Single fibres, isolated from EDL and gastrocnemius muscle, were analysed by confocal microscopy.

### Fibroblast and myoblast culture systems

Primary skin fibroblasts, derived from two unrelated patients with severe infantile PD, were obtained from Coriell Cell Repositories (Camden, New Jersey, USA). Clinical diagnosis was confirmed by genetic analysis. Fibroblasts were maintained in DMEM (high glucose) supplemented with 10% (v/v) FBS, 1× P/S/l-Glutamine. Adult mouse fibroblasts were isolated from the tail tip according to a standard procedure.

Immortalized PD cell lines were derived from immortoGAA−/− and immortoGFP-LC3:GAA−/− mice. Single fibres from EDL muscle of 3–4 month-old mice were plated in each well (∼10 fibres per well) of a Matrigel-coated six-well plate in plating medium (10% horse serum in DMEM, 0.5% chick embryo extract, and 1× P/S/l-Glutamine) at 37°C, 5% CO_2_. After 3 days, when the satellite cells began to migrate away from fibres, most of the medium (3/4) was replaced with proliferation medium (20% foetal bovine serum, 10% horse serum, 1% chick embryo extract, 1× P/S/l-Glutamine in high glucose DMEM). Myogenic colonies were isolated with cloning cylinders (Corning, Corning, NY) on day 5 or 6. Several individual myogenic clones were isolated and analysed for their proliferation capacity and the ability to differentiate into myotubes. To induce the expression of H-2K^b^-tsA58 SV40 large T-antigen for immortalization of the satellite cells and myoblasts, recombinant IFN-γ (Life Technologies) was added to the proliferation medium (100 units/ml). The cells were then incubated at 33°C in an atmosphere of 5% CO_2_ (Jat et al, 1991). When myoblasts became nearly confluent, the medium was changed to differentiation medium (DMEM containing 2% horse serum, 0.5% chick embryo extract, 1× P/S/l-Glutamine) and the cells were moved to 37°C in an atmosphere of 5% CO_2_. Myotubes began to form within 2 days. Glucose concentration was either 1 g/L (low) or 4.5 g/L (high). Myotubes survived in culture for 2–3 weeks. A wild type immortalized muscle cell line served as the control.

### Infection of myotubes with adenovirus expressing TFEB and immunofluorescence microscopy

Adenoviruses expressing either WT or mutant (S211A) TFEB were described previously (Martina et al, 2012). Myotubes were infected with either adenovirus (Ad-null) or adenovirus expressing WT TFEB (Ad-TFEB) or S211A TFEB (Ad-TFEBmt) for 24, 48 or 72 h. Myotubes were fixed in 2% paraformaldehyde (Electron Microscopy Sciences, Hatfield, PA) for 15 min at room temperature, washed twice in PBS, and permeabilized in 0.2% Triton X-100 (Sigma–Aldrich, St. Louis, MO). Immunostaining with LAMP1, LC3 or Flag antibodies was done using M.O.M. kit (Vector Laboratories, Burlingame, CA) as previously described (Raben et al, 2009). The cell nuclei were stained with 2 µg/ml Hoechst 33342 (Life Technologies) in PBS for 10 min. After staining, the cells were imaged on a Carl Zeiss LSM 510 confocal microscope with a 40× or 63× oil immersion objective.

### Fluorescent glycogen detection, LDH measurements, *in situ* detection of apoptotic cells, surface LAMP1 analysis, and acid phosphatase assay

Lysosomal glycogen in live cells was detected by the incorporation of 2-NBDG, a d-glucose fluorescent derivative (2-deoxyglucose), into glycogen as described (Louzao et al, 2008). The amount of LDH released into the medium was assayed using the LDH-Cytotoxicity Assay Kit II (Abcam) according to the manufacturer's instructions. Apoptotic cells were detected with TUNEL using the ApopTag® Fluorescein Direct *In Situ* Apoptosis Detection Kit (Millipore, Billerica, MA). Lysosomal exocytosis in TFEB-treated cell cultures was evaluated by the surface LAMP1 assay as previously described (Medina et al, 2011) and by the measurement of acid phosphatase released into the medium. The acid phosphatase assay was performed in cultures treated with Ad-TFEB for 2 days according to the standard procedure (Supporting Information, Table S1).

### Western blot

Cells were homogenized in RIPA buffer [PBS containing 1% NP-40, 0.5% sodium deoxycholate, 0.1% SDS and Protease/Phosphatase Inhibitor Cocktail (Cell Signaling Technology, Danvers, MA)] and centrifuged for 30 min at 13,000 rpm at 4°C. Alternatively, for nuclear/cytosolic fractionation, cells were lysed in 0.5% Triton X-100 buffer [50 mM Tris–HCl, 0.5% Triton X-100, 137.5 mM NaCl, 10% glycerol, 5 mM EDTA and protease and phosphatase inhibitors] and centrifuged at 3000 rpm at 4°C for 5 min. The supernatant constituted the cytosolic fraction. The pellet was then washed with 0.5% Triton X-100 buffer, re-suspended in Triton X-100 buffer with 0.5% SDS, sonicated, and centrifuged at 13,000 rpm for 15 min at 4°C. The supernatant constituted the nuclear fraction. Protein concentrations were measured using the Bio-Rad Protein Assay (Bio-Rad Laboratories, Inc., Hercules, CA). Western blots were performed according to standard procedures. Blots were scanned on an Odyssey Infrared Imager (LI-COR Biosciences, Lincoln, NE).

### Intramuscular injection of AAV-TFEB, muscle staining and glycogen assay, and electron microscopy (EM)

Six 1-month-old GAA−/− mice were injected with a total dose of 10^11^ GC of AAV2/1 vector preparation into three sites of the right gastrocnemius (three injections of 30 µl each) using a Hamilton syringe. Equivalent doses of AAV2/1CMV-EGFP or equal volumes of PBS were injected into the contralateral muscle. Animals were sacrificed 45 days after injection and perfused with PBS.

Gastrocnemii, free from neighbouring muscles and connective tissue, were isolated and analysed. Part of the sample was used to test the levels of TFEB expression by RT-PCR. For morphologic analysis, muscles were frozen in liquid nitrogen-cooled isopentane. Immunostaining of 10 µm sections was performed using anti-LAMP1 (1:500; Abcam, Cambridge, MA, USA) and anti-Caspase-3 Active (1:500; R&D Systems, Minneapolis, MN, USA) antibodies. Apoptotic cells were detected with TUNEL using the Fluorescein In Situ Cell Death Detection Kit (Roche, Basel, Switzerland) according to the supplier's protocol. Muscle sections were mounted with Vectashield, and photographs were taken using a fluorescence microscope Zeiss (Thornwood, NY) Axioplan 2 integrated with the AxioCam MR camera. Haematoxylin–eosin and PAS staining were performed according to standard procedures.

Glycogen concentration was assayed in muscle lysates by measuring the amount of glucose released after digestion with amyloglucosidase (*Aspergillus niger*) using a commercial kit (BioVision, Milpitas, CA, USA). Data were expressed as µg of glycogen/mg of protein. For EM, muscle tissue was fixed in 1% glutaraldehyde in 0.2 M HEPES buffer and post-fixed in uranyl acetate and OsO_4_. After dehydration through a graded series of ethanol and propilenoxide, the tissue was embedded in the Epoxy resin (Epon 812, Sigma–Aldrich) and polymerized at 60°C for 72 h. EM images of thin sections were acquired using a FEI Tecnai-12 electron microscope (FEI, Eindhoven, Netherlands) equipped with a VELETTA CCD digital camera (Soft Imaging Systems GmbH, Munster, Germany). Quantification of the number of lysosome-like organelles and their dimensions as well as the number of autophagosomes was performed in 50 fields (of 5 µm^2^ dimensions) distributed randomly through the thin sections containing different fibres.

Quantitative analyses are presented in the Supporting Information. Data in text are given as mean ± SE. Kolmogorov–Smirnov test was used for comparison of cumulative distributions of lysosomal size in myotubes and muscle fibres; Wilcoxon rank sum test was used for comparison of median values. Student's *t*-test was used for all other comparisons. Differences were considered significant at *p* < 0.05.

Animal care and experiments were conducted in accordance with the National Institutes of Health Guide for the Care and Use of Laboratory Animals and the European Union Directive 86/609 regarding the protection of animals used for experimental purposes.

## Author contributions

NR, EF, LL and JL performed tissue culture and live muscle fibre experiments, and analysed the data; RPu contributed new reagents and analytical tools, interpreted and analysed data; HZ performed statistical analysis and interpreted data; CS and FA generated vectors, performed intramuscular studies, and interpreted data; MC performed the biochemical and histological analyses of intramuscular studies; RPo performed the electron microscopy analysis; EF designed the quantitative analysis of the live fibre data and participated in writing and preparation of the manuscript; GP and AB designed and analysed the experiments and contributed to the writing of the paper; NR designed and analysed the experiments and wrote the paper.

## Notes

As a service to our authors and readers, this journal provides supporting information supplied by the authors. Such materials are peer reviewed and may be re-organized for online delivery, but are not copy-edited or typeset. Technical support issues arising from supporting information (other than missing files) should be addressed to the authors.
