# Supplementary material for: Transcription factor EB (TFEB) is a new therapeutic target for Pompe disease
Source: EMBO Mol Med. 2013 Apr 18;5(5):691–706. doi: 10.1002/emmm.201202176 (PMC3662313; doi:10.1002/emmm.201202176)
Supplement: Supplementary file 9 [file emmm0005-0691-sd9.pdf]

EMBO
Molecular Medicine

Transcription factor EB (TFEB) is a new therapeutic target for Pompe disease

Carmine Spampanato, Erin Feeney, Lishu Li, Monica Cardone, Jeong-A Lim, Fabio Annunziata, Hossein Zare, Roman Polishchuk, Rosa Puertollano, Giancarlo Parenti, Andrea Ballabio and Nina Raben

Corresponding authors: Andrea Ballabio, Telethon Institute of Genetics and Medicine (TIGEM) and Nina Raben, NIAMS, National Institutes of Health

---

Review timeline:

Submission date:	18 October 2012
Editorial Decision:	26 November 2012
Revision received:	30 January 2013
Editorial Decision:	15 February 2013
Revision received:	25 February 2013
Accepted:	27 February 2013

Transaction Report:

(Note: With the exception of the correction of typographical or spelling errors that could be a source of ambiguity, letters and reports are not edited. The original formatting of letters and referee reports may not be reflected in this compilation.)

Editor: Natascha Bushati / Céline Carret

1st Editorial Decision 26 November 2012

Thank you for the submission of your manuscript to EMBO Molecular Medicine. We have now heard back from the three referees whom we asked to evaluate your manuscript. You will see that they consider your manuscript to be of potential interest. However, they also raise significant concerns about the study, which would have to be addressed in a substantive revision of the manuscript.

Reviewer #1 highlights that additional data on the function of TFEB-treated Pompe diseased muscles is required to strengthen the significance of TFEB as a potential therapeutic target.

Importantly, reviewer #2 requires in-depth characterisation and quantification of exocytosis in myofibers overexpressing TFEB. In addition, this reviewer would like to see more careful characterisation of the observed apoptosis and an investigation into possible necrosis phenotypes. Finally, this reviewer is concerned about the observed mitochondrial changes and requests a more thorough analysis of the effect of TFEB transfection on mitochondria.

Reviewer #3 highlights the potential for toxicity of the proposed treatment, which we would ask you to address experimentally.

In our view the suggested revisions would render the manuscript much more compelling and interesting to a broad readership. We therefore hope that you will be prepared to undertake the recommended experimental revision.

Please note that it is EMBO Molecular Medicine policy to allow a single round of revision in order to avoid the delayed publication of research finding. Consequently, acceptance or rejection of the manuscript will depend on the completeness of your responses included in the next version of the manuscript.

Revised manuscripts should be submitted within three months of a request for revision. If your revision will have to exceed this time frame, please contact the editor. Please also contact the editor as soon as possible if similar work is published elsewhere.

I look forward to seeing a revised form of your manuscript in due course.

***** Reviewer's comments *****

Referee #1 (Comments on Novelty/Model System):

This is a well-written paper about application of TFEB expression as a therapeutic target for Pompe disease. TFEB expression gave successful results of clearance of antophagy build-up in myofibers to extracellular space. The authors also supply the excellent in vitro systems, as immortalized cell culture system and living single myofibers, which can be applicable for wider use, such as drug screening and in vitro testing before animal experiments.

Referee #1 (General Remarks):

The reviewer has only minor points.

1. The author said TFEB is new therapeutic target in title. The author showed that the clearance of autophagic build-up in myofibers well, but they did not supply the property of treated PD muscles. Providing information of the treated mouse muscles, motor performance, contractile force, muscle size and pathology, will help the readers to consider the therapeutic possibility of this treatment.
2. The authors showed the increase of the motility of lysosome after TFEB treatment. Beside of the importance of movement of lysosomes toward the plasma membrane for reducing their numbers in myofibers, what is the impact in increment of the velocity of lysosomes?
3. Discussion is too long. The first paragraph in Discussion, which is repetitive to Introduction, can be omitted.
4. TFEB expression solely could make a clearance of the accumulated glycogen with lysosomes from muscle fibers. It may indicate no longer necessity of ERT? If ERT still have a therapeutic impact with TFEB expression. Please discuss this point.

Referee #2 (Comments on Novelty/Model System):

The paper of N Raben group is extending to Pompe disease the findings of A Ballabio group about TFEB and lysosomal exocytosis. Indeed the findings are not novel. Ballabio group have already published that TFEB overexpression decrease glycogen content in fibroblast of Pompe patients (Figure 5D of Medina DL et al. 2011, Transcriptional Activation of Lysosomal Exocytosis Promotes Cellular Clearance. Dev. Cell. 21, 421-430). Even more important, the data of exocytosis are weak. Exocytosis has been superficially analysed. Moroever, TFEB expression show an effect on mitochondrial network that can explain per se the reduced glycogen loading in myofiber of GAA deficient mice. In fact, decrease of glycogen load in lysosome might be a consequence of a decrease glucose uptake or an increase of glucose utilization from uncoupled mitochondria. These aspects have not being considered by the authors. Altogether the paper in the present state is superficial and not enough novel to be published in EMBO Mol Med journal

Referee #2 (General Remarks):

The paper of N Raben group is extending to Pompe disease the findings of A Ballabio group about TFEB and lysosomal exocytosis. The authors have studied, in vitro, the potential contribution of

TFEB to clearance of glycogen filled lysosomes by using adult myofiber and myoblast obtained from GAA knockout mice. The overexpression of TFEB in myotubes and myofibers ameliorates the glycogen load and decrease the size of lysosomes. By using different tools the authors have also shown that TFEB ameliorates autopahgosome-lysosome fusion and induces lysosome exocytosis. Finally they extend in vivo the results obtained in cell culture by overexpressing FTEB in skeletal muscles of GAA knockout mice. The overall paper is well written but the findings are not novel. Indeed, Ballabio group have already published that TFEB overexpression decrease glycogen content in fibroblast of Pompe patients (Medina DL et al. 2011, Transcriptional Activation of Lysosomal Exocytosis Promotes Cellular Clearance. Dev. Cell. 21, 421-430). The authors should consider the following points.

Point 1. Lysosomal exocyotsis is the main issue of this paper. However all the findings that support exocytosis are based on imaging system to detect relocation of Lamp1 positive vesicles from the cytoplasm of myotube to plasma membrane. However, Lamp1-cherry has been never shown on the plasma membrane of GAA-/- myofibers that overexpress TFEB. The authors must better characterize that truly exocytosis happens in their condition. Release of lysosomal enzymes (acid phosphatase, b-galactosidase, b-exosaminidase) have to be shown to occur in TFEB overepressing myofibers. Moreover, the most important piece of data is the clearance of glycogen-filled lysosomes in GAA-/- myofibers. The release of glycogen in the cell culture medium must be studied. Furthermore, exocytosis inhibitors should be used to block the beneficial effect of TFEB expression on glycogen load. Finally the authors should use EM to show the lysosomal/autophagosomal exocytosis event in TFEB overexpressing fibers.

Point 2. Lamp1 is sorted to lysosomes via endosomes pathways. The authors should monitor whether TFEB overexpression affect endosome trafficking that place Lamp1-cherry in the secretory pathways via Tubular Sorting Endosome.

Point 3. Fig. 3B. There re two bands and the lower one is indeed increased in ad-TFEBmut samples. Explain why two bands and whether this increase is significant and what it means.

Point 4. Fig 3C. TUNEL technique can not distinguish necrosis from apoptosis unless it is coupled with morphological analyses and caspase 3/6/7 activation. Moreover the blebbing present in TFEB-overexpressing fibers is also an index of necrosis or necroptosis. Authors must characterized better apoptosis by monitoring caspase 3 activation and chromatin fragmentantion and condensation. Moreover also necrosis should be studied by determining LDH release, propidium iodide incorporation or membrane permeabilization (Evans blue uptake), RIP1/3 accumulation.

Point 5. Fig. S5A, B). The figure of autophagy activation in Torin1/2 treated cells are too small to sustain authors claim. Please show higher magnification in order to reveal LC3 positive puncta and quantify the increase of vesicles. Alternatively show LC3 lipidation by western blotting. The authors claim: "A striking increase in the levels of p-ERK1/2 in TFEB-treated PD myotubes points to such a possibility in muscle cells" is incorrect. The data are instead supporting the concept that ERK1/2 are downstream TFEB. To sustain their claim the authors should block ERK by treting cells with inhibitors (or knockdown ERK) .

Point 6. The authors showed an increase of lysosome motility and fusion by TFEB overexpression in myofibers of GAA knockout mice. They also found that the number of large lysosome is reduced. However the movies and the pictures also show the appearance of huge lysosomes that came out from the fusion of enlarged lysosomes. Therefore, it looks like that TFEB induces the fusion of lysosome into giant vacuoles. Please quantify and show the distribution of lysosome sizes in control and TFEB overexpressing fibers.

Point 7. Fig 4B and movies. Most of the time TFEB overexpression induces the formation of blebs, The blebs are index of cellular swelling and therefore of pre-necrotic events (Fig 4B, Fig 6B, Movie 1, Movie 6). Necrosis should be studied (see above point 4). Moreover, many movies of TFEB overexpressing fibers show movement of myofibers that causes changes of focus and area of observation (fiber become larger or smaller) (Movie 1, Movie 6, Movie 7). Therefore, it is very difficult to follow vesicles trafficking and fusion events and to interpret the data. Movie 8 is not working.

Point 8. Fig. 8 A and B. Mitochondria morphology and distribution looks different in TFEB overexpressing muscles. Indeed the mitochondria localized nearby the Z-line (the correct localization) are bigger in panel B (TFEB transfected muscle). Conversely the accumulation of big and abnormal mitochondria closed to giant lysosomes that is depicted in panel A is absent in panel B. Is TFEB affecting mitochondria number, morphology and localization? In the case of an increase of mitochondria, are mitochondria uncoupled? Is glucose and lipid homeostasis altered? In fact, decrease of glycogen load in lysosome might be a consequence of a decrease glucose uptake or an increase of glucose utilization from uncoupled mitochondria. All these conditions must be analyzed in order to give a clear picture of TFEB action in muscles.

Minor points:

The authors often use the words Lamp1 and Lamp as synonyms. This is wrong since it exists also Lamp2 proteins that have different functions (CMA) from Lamp1.

Referee #3 (Comments on Novelty/Model System):

This is an outstanding paper. The only reservation is with regard to the potential medical impact, because the treatment might have unsuspected toxicity.

Referee #3 (General Remarks):

This is an outstanding manuscript describing application of a new therapeutic strategy to Pompe disease.

Major concerns:

1) The potential for toxicity from induction of apoptosis in PD muscle is high. It would be reassuring to know that toxicity in TFEB-treated mice was absent (central nuclei, elevated transaminases or creatine kinase) in AAV experiment.
2) Lacking the information in response to my first concern, it would be better to highlight the potential for toxicity in vivo by addressing this subject in the discussion. A long-term experiment that addressed these questions would be the only definitive response, and that might not be available for the current manuscript. Therefore the possibility of unexpected toxicity must be addressed in the discussion.

Minor concerns:

1) Abstract: Why is Glycogen Storage Disease capitalized?
2) Page 5, paragraph 3: "studying the lysosomal defect" would be correct.
3) Page 5, paragraph 4: "with an adenovirus vector expressing" would be correct.

1st Revision - authors' response 30 January 2013

POINT-BY-POINT RESPONSE TO THE REVIEWERS' COMMENTS

Referee #1 (General Remarks):

The reviewer has only minor points.
1. The author said TFEB is new therapeutic target in title. The author showed that the clearance of autophagic build-up in myofibers well, but they did not supply the property of treated PD muscles. Providing information of the treated mouse muscles, motor performance, contractile force, muscle size and pathology, will help the readers to consider the therapeutic possibility of this treatment.

© EMBO

We have addressed the point raised by the reviewer experimentally, and the data have been included in the revised manuscript. According to the reviewer's suggestions we evaluated fiber pathology of treated muscles. Hematoxylin-eosin staining did not show gross alterations of the muscular architecture in TFEB-treated gastrocnemii, compared to untreated muscles, or signs of toxicity (Supporting information; Figure S9 A). No differences were seen in the number of centralized nuclei in treated and untreated muscles. TUNEL and caspase 3 staining of muscle preparations from TFEB-treated muscles did not show an increase of apoptotic cells (Supporting information, Figure S9 B and C). Muscle size was also evaluated and no significant differences were observed between untreated and TFEB-treated muscles (Supporting information, Figure S9 D and E).

2. The authors showed the increase of the motility of lysosome after TFEB treatment. Beside of the importance of movement of lysosomes toward the plasma membrane for reducing their numbers in myofibers, what is the impact in increment of the velocity of lysosomes?

As the reviewer correctly points out, an increase in lysosomal "dynamics" may impact not only on lysosomal exocytosis but may have more general consequences on intracellular trafficking. For example, we have previously demonstrated that TFEB enhances the fusion between lysosomes and autophagosomes (Settembre et al. Science 2011). However, while we recognize the importance of this point, it would be very difficult, and out of the scope of this paper, to determine the overall impact in increment of the velocity of lysosomes.

3. Discussion is too long. The first paragraph in Discussion, which is repetitive to Introduction, can be omitted.

We shortened the discussion in the revised manuscript.

4. TFEB expression solely could make a clearance of the accumulated glycogen with lysosomes from muscle fibres. It may indicate no longer necessity of ERT? If ERT still have a therapeutic impact with TFEB expression. Please discuss this point.

The reviewer is right: this is a very important point. We added comments regarding a possible application of a combined approach (ERT + TFEB) to the Discussion.

Referee #2 (Comments on Novelty/Model System):

The paper of N Raben group is extending to Pompe disease the findings of A Ballabio group about TFEB and lysosomal exocytosis. Indeed the findings are not novel. Ballabio group have already published that TFEB overexpression decrease glycogen content in fibroblast of Pompe patients (Figure 5D of Medina DL et al. 2011, Transcriptional Activation of Lysosomal Exocytosis Promotes Cellular Clearance. Dev. Cell. 21, 421-430). Even more important, the data of exocytosis are weak. Exocytosis has been superficially analysed. Moreover, TFEB expression show an effect on mitochondrial network that can explain per se the reduced glycogen loading in myofiber of GAA deficient mice. In fact, decrease of glycogen load in lysosome might be a consequence of a decrease glucose uptake or an increase of glucose utilization from uncoupled mitochondria. These aspects have not being considered by the authors. Altogether the paper in the present state is superficial and not enough novel to be published in EMBO Mol Med journal.

Referee #2 (General Remarks):

The paper of N Raben group is extending to Pompe disease the findings of A Ballabio group about TFEB and lysosomal exocytosis. The authors have studied, in vitro, the potential contribution of TFEB to clearance of glycogen filled lysosomes by using adult myofiber and myoblast obtained from GAA knockout mice. The overexpression of TFEB in myotubes and myofibers ameliorates the

© EMBO

glycogen load and decrease the size of lysosomes. By using different tools the authors have also shown that TFEB ameliorates autopahgosome-lysosome fusion and induces lysosome exocytosis. Finally they extend in vivo the results obtained in cell culture by overexpressing FTEB in skeletal muscles of GAA knockout mice. The overall paper is well written but the findings are not novel. Indeed, Ballabio group has already published that TFEB overexpression decrease glycogen content in fibroblast of Pompe patients (Medina DL et al. 2011, Transcriptional Activation of Lysosomal Exocytosis Promotes Cellular Clearance. Dev. Cell. 21, 421-430). The authors should consider the following points.

We disagree with the reviewer's opinion on the lack of novelty of our paper for the following reasons:

1. If one wants to test any new therapeutic approach to Pompe disease, the most relevant systems to use are multinucleated muscle cells, muscle fibers, and whole muscle, the tissue that is phenotypically affected and most difficult to treat by the currently available drug. We have used all three systems. The data on TFEB in Pompe disease that we published in a previous study (Medina et al. Dev. Cell 2011) were extremely limited (one figure subpanel). Indeed they consisted of preliminary experiments in fibroblasts from <u>a single patient</u>. No data on muscle, which is the major site of pathology, and no *in vivo* data from Pompe mouse models were previously published. Therefore, all <u>findings</u> in the current manuscript are novel.

2. We feel that the Referee overlooked the importance of the data on autophagy, in particular the results of TFEB expression in autophagy-deficient mice. The idea that functional autophagy is required for fully efficient TFEB-mediated clearance is an entirely <u>new concept.</u>

Point 1. Lysosomal exocytosis is the main issue of this paper. However all the findings that support exocytosis are based on imaging system to detect relocation of Lamp1 positive vesicles from the cytoplasm of myotube to plasma membrane. However, Lamp1-cherry has been never shown on the plasma membrane of GAA-/- myofibers that overexpress TFEB. The authors must better characterize that truly exocytosis happens in their condition. Release of lysosomal enzymes (acid phosphatase, b-galactosidase, b-exosaminidase) has to be shown to occur in TFEB overexpressing myofibers. Moreover, the most important piece of data is the clearance of glycogen-filled lysosomes in GAA-/- myofibers. The release of glycogen in the cell culture medium must be studied. Furthermore, exocytosis inhibitors should be used to block the beneficial effect of TFEB expression on glycogen load. Finally the authors should use EM to show the lysosomal/autophagosomal exocytosis event in TFEB overexpressing fibers.

We have added the data on the release of lysosomal acid phosphatase in the medium following TFEB treatment of myotubes (Supporting information, Table S1). These data, combined with the appearance of LAMP1 on the cell surface, provide strong evidence of lysosomal exocytosis (as now emphasized in the revised manuscript). As for the glycogen measurement in culture medium, the biochemical assay for glycogen (which is not terribly sensitive in the first place) is based on the conversion of glycogen to glucose, followed by glucose measurement. Myotubes grow and differentiate in the medium with high concentration of glucose, which precludes accurate measurements due to the high background. The data in myotubes strongly suggest that TFEB induces lysosomal exocytosis in muscle. In addition, we tried to measure lysosomal exocytosis in live TFEB-transfected fibers but soon realized that the separation of transfected from non-transfected fibers (a procedure required for any comparison) is not feasible, particularly given the relatively low transfection efficiency. Picking up only transfected fibers (which are surrounded by huge numbers of non-transfected fibers) and subsequently exposing them to multiple rounds of re-plating invariably results in fiber contraction.

Point 2. Lamp1 is sorted to lysosomes via endosomes pathways. The authors should monitor whether TFEB overexpression affect endosome trafficking that place Lamp1-cherry in the secretory pathways via Tubular Sorting Endosome.

In Pompe disease glycogen accumulates in hugely enlarged Lamp1-positive structures. It is well known that these structures are lysosomes and not tubular sorting endosomes, which do not

accumulate glycogen. In addition, in a previous study we demonstrated that TFEB overexpression has no effect on endocytic secretory pathways (Medina et al. Dev Cell 2011).

Point 3. Fig. 3B. There are two bands and the lower one is indeed increased in ad-TFEBmut samples. Explain why two bands and whether this increase is significant and what it means.

LAMP1 never runs as a single band on western; additional bands most likely represent different glycosylation forms of the protein. The increase in LAMP1 in TFEB-treated cells is not unexpected since TFEB was shown to stimulate lysosomal biogenesis. We have added this information to the revised manuscript.

Point 4. Fig 3C. TUNEL technique cannot distinguish necrosis from apoptosis unless it is coupled with morphological analyses and caspase 3/6/7 activation. Moreover the blebbing present in TFEB-overexpressing fibers is also an index of necrosis or necroptosis. Authors must characterized better apoptosis by monitoring caspase 3 activation and chromatin fragmentation and condensation. Moreover also necrosis should be studied by determining LDH release, propidium iodide incorporation or membrane permeabilization (Evans blue uptake), RIP1/3 accumulation.

We have followed the reviewers' suggestions and measured LDH release and caspase-3 activity. No signs of apoptosis or necrosis were detected according to these methods. This information was added to the paper. As for the blebbing in TFEB-overexpressing fibers, we would like to emphasize that this phenomenon is not seen in freshly isolated fibers or fibers maintained for days in culture. The blebbing is only seen when the fibers are exposed for hours to time-lapse confocal microscopy. We say this very clearly in the revised manuscript.

Point 5. Fig. S5A, B). The figure of autophagy activation in Torin1/2 treated cells is too small to sustain authors claim. Please show higher magnification in order to reveal LC3 positive puncta and quantify the increase of vesicles. Alternatively show LC3 lipidation by western blotting. The authors claim: "A striking increase in the levels of p-ERK1/2 in TFEB-treated PD myotubes points to such a possibility in muscle cells" is incorrect. The data are instead supporting the concept that ERK1/2 are downstream TFEB. To sustain their claim the authors should block ERK by treating cells with inhibitors (or knockdown ERK) .

We provide a new figure S5 with insets for enlarged views and with additional data on LC3. We have also modified the discussion to address the referee's suggestion regarding ERK.

Point 6. The authors showed an increase of lysosome motility and fusion by TFEB overexpression in myofibers of GAA knockout mice. They also found that the number of large lysosome is reduced. However the movies and the pictures also show the appearance of huge lysosomes that came out from the fusion of enlarged lysosomes. Therefore, it looks like that TFEB induces the fusion of lysosome into giant vacuoles. Please quantify and show the distribution of lysosome sizes in control and TFEB overexpressing fibers.

We have added the graph on distribution of lysosomal sizes in control and TFEB-overexpressing fibers. The difference between the two conditions is highly significant.

Point 7. Fig 4B and movies. Most of the time TFEB overexpression induces the formation of blebs, The blebs are index of cellular swelling and therefore of pre-necrotic events (Fig 4B, Fig 6B, Movie 1, Movie 6). Necrosis should be studied (see above point 4). Moreover, many movies of TFEB overexpressing fibers show movement of myofibers that causes changes of focus and area of observation (fiber become larger or smaller) (Movie 1, Movie 6, Movie 7). Therefore, it is very difficult to follow vesicles trafficking and fusion events and to interpret the data. Movie 8 is not working.

Regarding “blebbing” see point 4 above. It is no surprise that the fibers may move a bit, and indeed it is tricky to track vesicular movements. Time-lapse microscopy of live fibers is in general not a trivial endeavour. Suffice it to say that during each session, out of ~30 selected fibers, only one or two fibers (and often none) survive. However, when we analyse the fibers for vesicle velocity, we very carefully select the fibers and the time points with minimal twitching. This information was included in the original manuscript (Supporting Information): “To minimize the contribution of fiber movement to lysosomal velocity measurements, manual tracking was performed only between time points when fiber twitching was not ostensible.”
We fixed Movie 8 – thank you very much for pointing to the problem.

Point 8. Fig. 8 A and B. Mitochondria morphology and distribution looks different in TFEB overexpressing muscles. Indeed the mitochondria localized nearby the Z-line (the correct localization) are bigger in panel B (TFEB transfected muscle). Conversely the accumulation of big and abnormal mitochondria closed to giant lysosomes that is depicted in panel A is absent in panel B. Is TFEB affecting mitochondria number, morphology and localization? In the case of an increase of mitochondria, are mitochondria uncoupled? Is glucose and lipid homeostasis altered? In fact, decrease of glycogen load in lysosome might be a consequence of a decrease glucose uptake or an increase of glucose utilization from uncoupled mitochondria. All these conditions must be analysed in order to give a clear picture of TFEB action in muscles.

We agree with the reviewer on the fact that TFEB may have an effect on glucose/glycogen metabolism. However, the fibers are isolated and analysed 4 days after TFEB transfection, and any "short term" change in glucose/glycogen metabolism would have negligible effects on muscle glycogen accumulation, which takes much longer to build in Pompe disease.
The Referee also raises an interesting question about mitochondria in general. We have looked at mitochondria using MitoTracker in live fibers and immunostaining of fixed fibers with cytochrome c. We found no difference in the mitochondrial distribution and abundance in control and TFEB - treated fibers.

As for the EM analysis, the purpose of Figure 8 is to show the striking reduction in size and number of glycogen-filled lysosomes. We provide additional images (see below) to the point-by-point response. These show that big mitochondria (arrows in panel A, left) can also be seen in preparations from untreated muscles.

The other concern raised by the reviewer is the accumulation of giant mitochondria around lysosomes in untreated muscle. We regularly see hugely enlarged lysosomes without adjacent clusters of mitochondria (arrowhead in panel A, right) in untreated samples. Conversely, we could see accumulation of mitochondria (arrows in panel B, left) near giant lysosomes in TFEB-injected mice. This illustrates the heterogeneity of muscle tissue rather than a strict pattern of mitochondrial distribution.
To address the reviewer’s concerns about the morphology of mitochondria we performed additional analyses. Mitochondria in treated and untreated muscles look quite similar in terms of size, cristae morphology, and electron density. A quantitative analysis of the number and size of mitochondria has now been added (Figure 8 C).

It is worth noting, though, that such accumulations of mitochondria near lysosomes in TFEB-expressing muscles are less prominent likely due to the increased mitophagy. Indeed, the only major difference in the appearance of mitochondria between control and TFEB-injected mice was the presence of mitochondria inside an autophagosome (see panel C). We think however that increased mitophagy is just a consequence of general increase in autophagy triggered by TFEB overexpression rather than a result of the mitochondria dysfunction.

Attachment

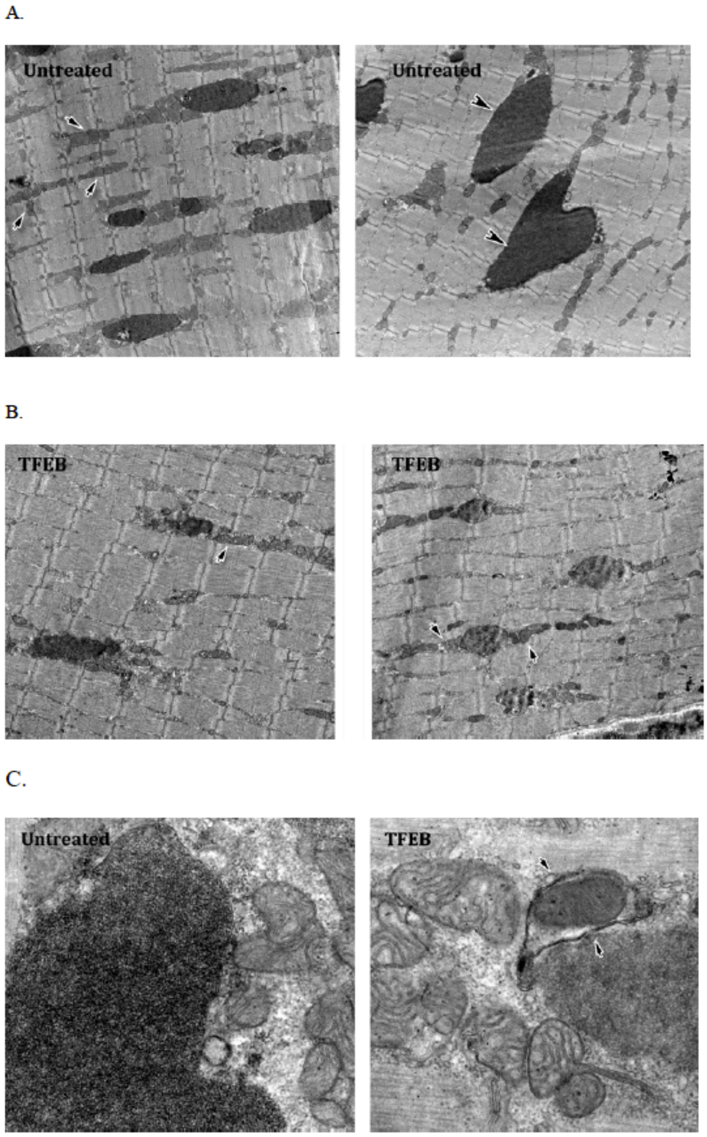

Minor points:

The authors often use the words Lamp1 and Lamp as synonyms. This is wrong since it exists also Lamp2 proteins that have different functions (CMA) from Lamp1.

We now consistently use LAMP1 instead of Lamp.

Referee #3 (Comments on Novelty/Model System):

This is an outstanding paper. The only reservation is with regard to the potential medical impact, because the treatment might have unsuspected toxicity.

Referee #3 (General Remarks):

This is an outstanding manuscript describing application of a new therapeutic strategy to Pompe disease.

Major concerns:

1) The potential for toxicity from induction of apoptosis in PD muscle is high. It would be reassuring to know that toxicity in TFEB-treated mice was absent (central nuclei, elevated transaminases or creatine kinase) in AAV experiment.
2) Lacking the information in response to my first concern, it would be better to highlight the potential for toxicity in vivo by addressing this subject in the discussion. A long-term experiment that addressed these questions would be the only definitive response, and that might not be available for the current manuscript. Therefore the possibility of unexpected toxicity must be addressed in the discussion.

No signs of toxicity were seen in standard H&E preparations. No abnormalities were observed by TUNEL assay and caspase 3 staining (see response to reviewer 1, point 1 and Supporting information Figure S9 B and C).

Minor concerns:

1) Abstract: Why is Glycogen Storage Disease capitalized?
2) Page 5, paragraph 3: "studying the lysosomal defect" would be correct.
3) Page 5, paragraph 4: "with an adenovirus vector expressing" would be correct.

We accepted the editing – thank you.

2nd Editorial Decision 15 February 2013

Thank you for the submission of your revised manuscript to EMBO Molecular Medicine. We have now received the enclosed reports from the referees that were asked to re-assess it. As you will see the reviewers are now globally supportive and I am pleased to inform you that we will be able to accept your manuscript pending the following final amendments:

As you can see from the Referee's reports below, reviewer 1 remains concerned about the muscle properties after treatment and wondered whether the lack of symptoms could not be due to mice being too young to be symptomatic. As we feel that this is a valid concern, we would like you to acknowledge this possibility in the discussion part of your study.

I look forward to seeing a new revised version of your manuscript as soon as possible.

***** Reviewer's comments *****

Referee #1 (Comments on Novelty/Model System):

The model mice the authors used are too young to show the symptomatic phenotype.

Referee #1 (General Remarks):

The authors answered to almost points well, however the reviewer feels a bit of concern only about therapeutic impact of this treatment. The authors showed a clearance of lysosomes with glycogen from GAA-/- mice myofibers. Even if those lysosomal build-ups are biomarker of this disease, the authors should provide the significance of improvement of muscle properties in their mice after treatment. The authors said no difference in muscle pathology before and after treatment, as probably this is due to the age of mice, 2.5 months old at an experimental end point, as presymptomatic age.

Referee #2 (Comments on Novelty/Model System):

The paper still lack of novelty. It is an extension of the previous published data to Pompe disease. The medical impact is also medium since activation of endogenous TFEB does not induce any effect on glycogen clearance. Overexpression of TFEB by using virus is dangerous and not feasible as therapeutic approach.

Referee #2 (General Remarks):

The authors have addressed most of my concerns and the paper is greatly improved.

2nd Revision - authors' response 25 February 2013

POINT-BY-POINT RESPONSE TO THE REVIEWERS' COMMENTS

Referee #1:

The reviewer has only minor points.

Referee #1 (General 0Remarks):

The authors answered to almost points well, however the reviewer feels a bit of concern only about therapeutic impact of this treatment. The authors showed a clearance of lysosomes with glycogen from GAA-/- mice myofibers. Even if those lysosomal build-ups are biomarker of this disease, the authors should provide the significance of improvement of muscle properties in their mice after treatment. The authors said no difference in muscle pathology before and after treatment, as probably this is due to the age of mice, 2.5 months old at an experimental end point, as presymptomatic age.

The reviewer is right- the knockout mice develop clinical phenotype (profound muscle weakness and wasting, kyphosis, etc.) late, at the age of 7-9 months. The studies in older mice are needed to evaluate the effect of TFEB overexpression on muscle function. We acknowledge the need for pre-clinical studies to fully evaluate the consequences of TFEB activation.
