# Supplementary material for: Transcription factor EB (TFEB) is a new therapeutic target for Pompe disease
Source: EMBO Mol Med. 2013 Apr 18;5(5):691–706. doi: 10.1002/emmm.201202176 (PMC3662313; doi:10.1002/emmm.201202176)
Supplement: Supplementary file 10 [file emmm0005-0691-sd10.pdf]

Supplementary Information for

"Transcription factor EB (TFEB) is a new therapeutic target for Pompe disease"

Table of Contents

Figure S1. **Characterization of PD immortalized myoblast cell lines……………......2-3**

Figure S2. **Lysosomes and glycogen in patients' fibroblasts………………………….... . 4**

Figure S3. **Immortalized cell lines derived from GFP-LC3:GAA-/- fail to replicate autophagic pathology.......……………………………………….... 5**

Figure S4. **TFEB promotes cellular clearance and stimulates autophagy in PD myotubes……………………………………………………………….6**

Figure S5. **Torin 2 (an mTORC 1 inhibitor) stimulates autophagy, but not lysosomal clearance in PD myotubes……………………………………7**

Figure S6. **TFEB promotes clearance of enlarged lysosomes in PD fibers…………. ..8**

Figure S7. **TFEB expression in PD fibers does not affect mitochondrial distribution and abundance.…………………………………………………9**

Figure S8. **Occasional TFEB- transfected fibers exhibit membrane detachment**……………………………………………………………**10**

Figure S9. TFEB expression does not result in any appreciable abnormalities in PD muscle……………………………………………………………………. 11

Table S1. **Acid phosphatase assay in non-treated and Ad-TFEB-treated myotubes…12**

Equipment and settings………………………………………………………………. ..13

Additional Methods…………………………...……………………………………. 14-15

Movie captions………………………………………………………………………..16

References………………………………………………………………………………. 17

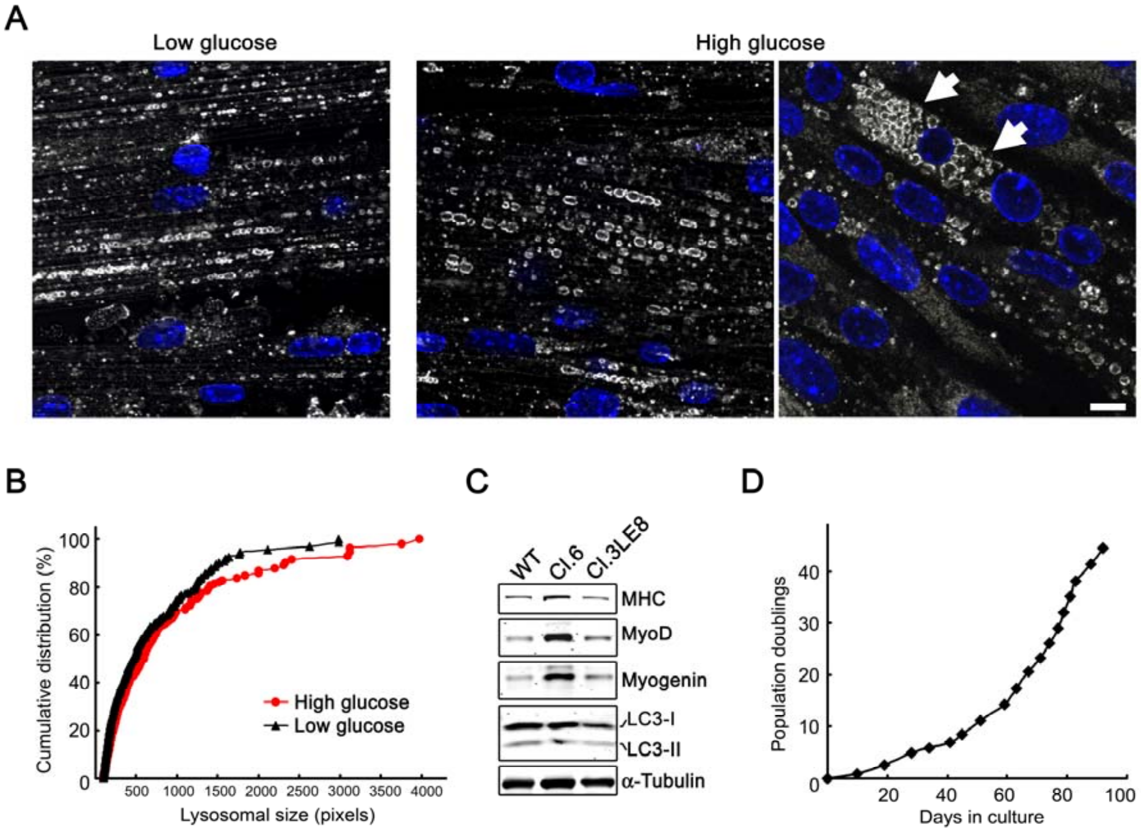

Figure S1. Characterization of PD immortalized myoblast cell lines.

Three myogenic cell lines –cl.6, cl.LA3, and cl. 3LE8– were chosen for further analysis (the data for cl.6 and cl.3LE8 are shown). These cells were derived from immortoGAA-/- mice, which express the temperature-sensitive SV40 large T antigen tsA58 under the control of interferon-gamma inducible murine H2Kb promoter. The myoblasts undergo immortalization when grown at 33°C with interferon-γ; the differentiation into multinucleated myotubes proceeds when the oncogene is silenced (37° C in the absence of interferon-γ). (**A**) Lysosomes were stained with LAMP1 (white); nuclei were stained with Hoechst. Lysosomal enlargement was observed starting at day 5-7 in differentiation medium. Myotubes grown in medium with high glucose concentration (4.5mg/L) contained larger lysosomes (up to ~10 microns by day 8-10) compared to those grown in medium with low glucose (1.0mg/L; ~5-7 microns by day 12). Clusters of swollen lysosomes with compromised membranes (arrows) suggesting lysosomal rupture – a feature typically found in muscle biopsies from severely affected infants (Raben et al, 2010a; Thurberg et al, 2006) - are seen in some myotubes (right). Myotubes were defined as cells with more than three nuclei. The fusion index, calculated as the percentage of nuclei in multinucleated cells, reached > 90% after 48 hours in differentiation medium. Bar: 10 μm. (**B**) Graphical presentation

of data in **A**. (**C)** Expression of myogenin, MyoD and Myosin Heavy Chain (MHC) was monitored by Western analysis. (**D)** The cell lines maintain the ability to differentiate after multiple passages (>35 population doubling). Growth curve for clone 3LE8 is shown.

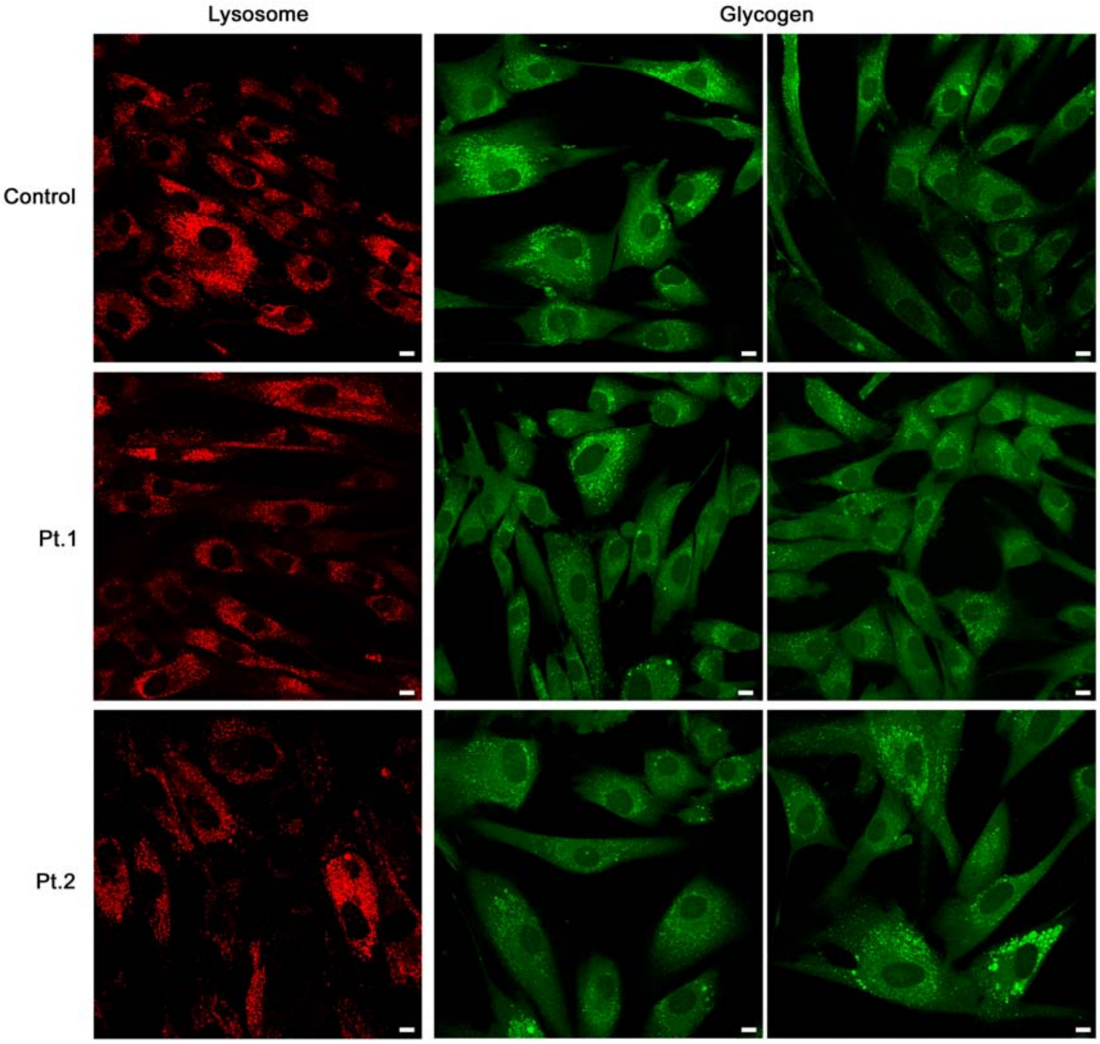

Figure S2. Lysosomes and glycogen in patients' fibroblasts.

Fibroblasts from an unaffected control individual and from two PD patients with the infantile form of the disease were stained for LAMP (red). The cells were also analyzed for glycogen content by loading with fluorescent glucose (2-NBDG; green) followed by confocal microscopy. The images show little difference in the lysosomal size and glycogen content in diseased compared to normal cells, although occasional PD cells (as well as occasional wt cells) may exhibit the phenotype. Similar results were obtained in mouse embryonic and adult fibroblasts. Bar: 10μm.

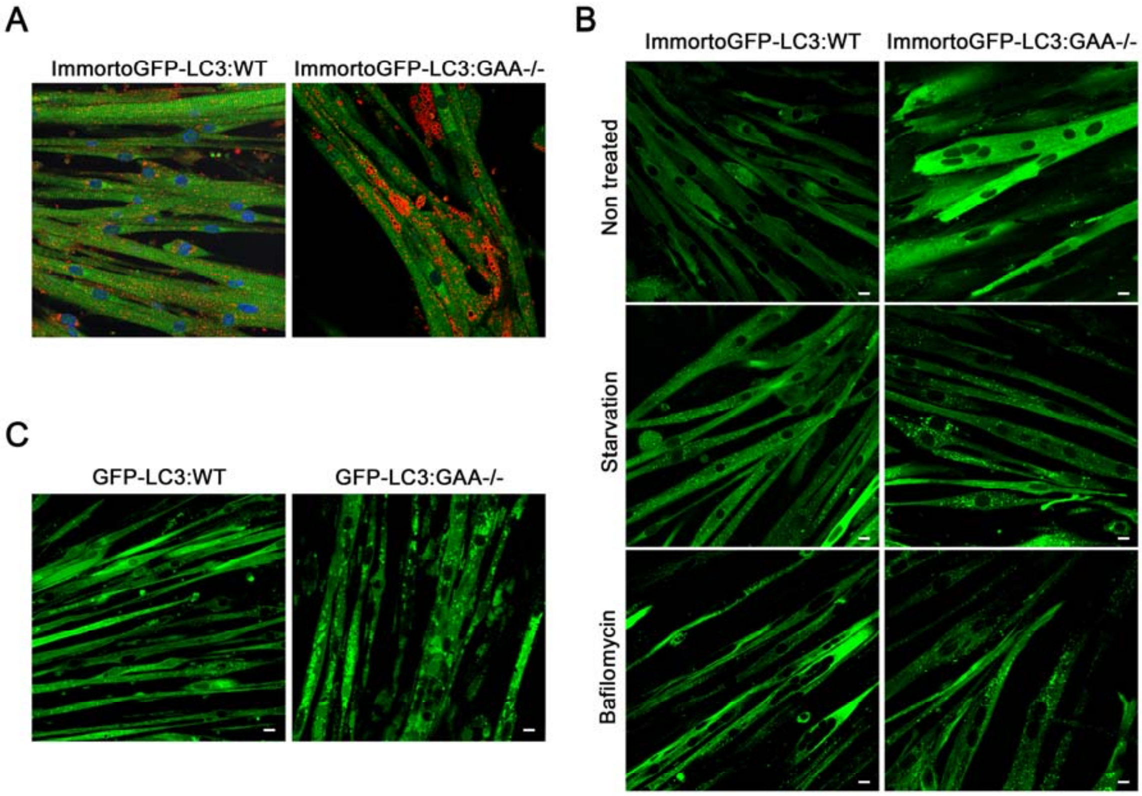

Figure S3. Immortalized cell lines derived from GFP-LC3:GAA-/- mice fail to replicate autophagic pathology.

In an attempt to uncover any subtle abnormalities in autophagy in our cell culture system, we made immortalized myoblast cell lines from GFP-LC3:GAA-/- mice. **(A)** Myotubes expressing GFP-LC3 were fixed and stained for LAMP (red). Nuclei are stained with Hoechst (blue). ImmortoGFP-LC3:GAA-/- myotubes contain enlarged lysosomes. The levels of GFP-LC3 expression are no different in PD cells compared to controls. (**B)** Both immortoGFP-LC3:WT and immortoGFP-LC3:GAA-/- cells exhibit functional autophagy, as shown by the response to starvation (4 hours in Krebs/Ringer's buffer) and bafilomycin (4 hours; 400 nM). (**C)** Primary myotubes from a GFP-LC3:GAA-/- mouse showed multiple clusters of LC3-positive vesicular structures (reminiscent of autophagic buildup in PD muscle) after 59 days in culture. WT myotubes showed diffuse LC3 expression with sparse dot-like structures. Live unstained samples were analyzed by confocal microscopy. Bar: 10 µm.

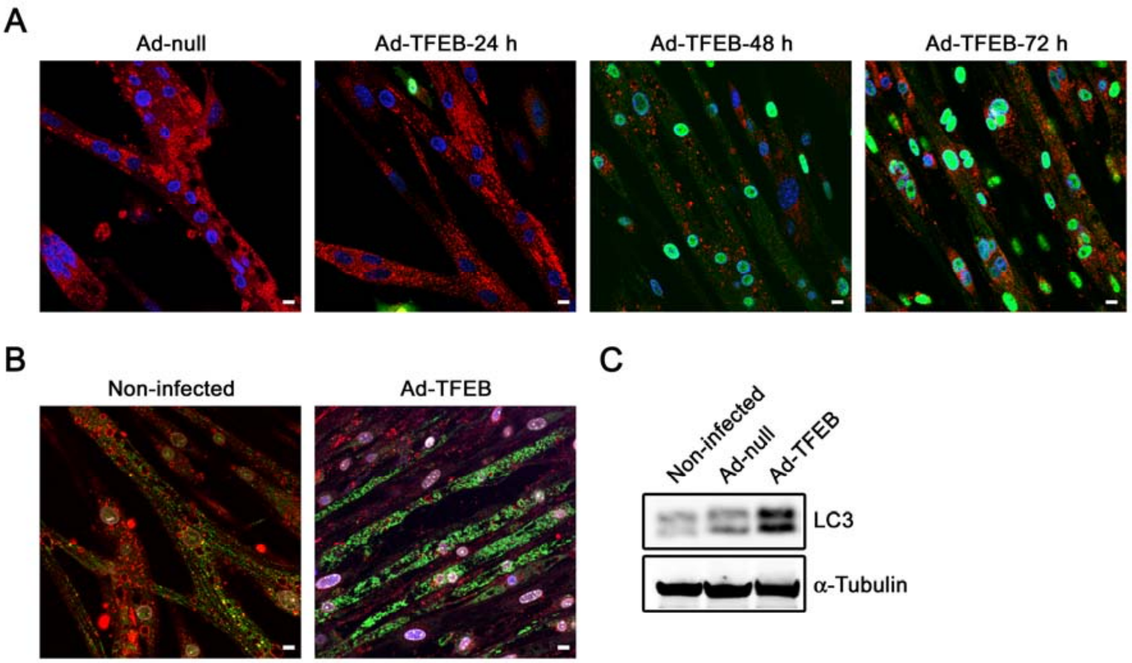

Figure S4. TFEB promotes cellular clearance and stimulates autophagy in PD myotubes.

(A) Time course of TFEB expression in infected PD myotubes. By 24 hours post-infection most myoblasts (green), but not myotubes express TFEB. By 48 hours, the majority of the cells express TFEB; by 72 hours, virtually all myotubes express TFEB. Ad-TFEB was added on day 10 after the differentiation began. Ad-null infected or TFEB-infected cells were then fixed and stained for LAMP (red) and Flag (green). Nuclei were stained with Hoechst (blue). (**B)** Non-infected and TFEB-infected PD myotubes were grown in differentiation medium for 9 days before being fixed and stained with LAMP1 (lysosomes; red), LC3 (autophagosomes; green), and Flag (TFEB; blue) antibodies. Nuclei are stained with Hoechst (white). (**C**) Western blotting of lysates obtained from non-infected cells and cells infected with ether adenovirus (Ad-null) or adenovirus containing TFEB (Ad-TFEB) using LC3 antibodies. α-Tubulin was used as a loading control. At least three to four experiments with each of the two clones were performed. Bar: 10 µm.

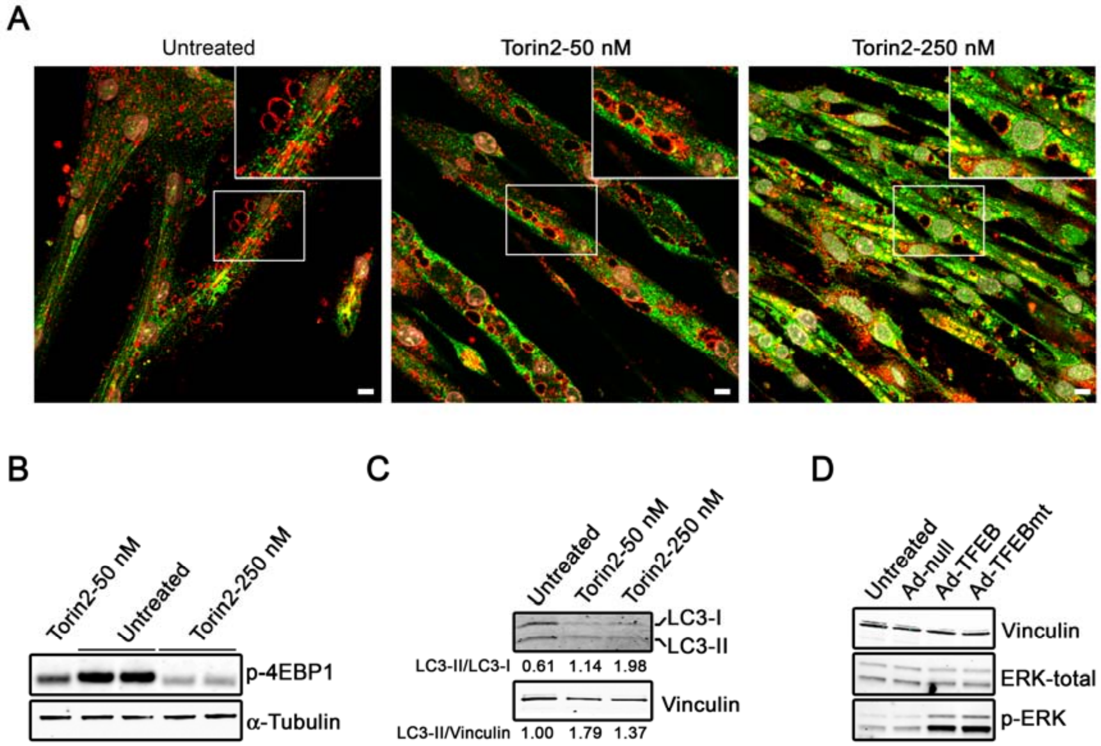

Figure S5. Torin 2 (an mTORC 1 inhibitor) stimulates autophagy, but not lysosomal clearance in PD myotubes.

(A) Immunostaining of untreated and Torin2-treated PD myotubes with LAMP (red), and LC3 (green). Nuclei were stained with Hoechst (blue). All cells were analyzed on day 12 after differentiation began; 50nM or 250nM of Torin 2 was added on day 9 for 3 days. Bar: 10 µm. (**B)** Western blotting of lysates obtained from untreated and Torin2-treated myotubes using 4-EBP1 antibody. α-Tubulin was used as a loading control. Inhibition of mTORC 1 is indicated by a decreased phosphorylation of its downstream target, 4-EBP1. (**C**) Western blotting of lysates obtained from untreated and Torin2-treated myotubes using LC3 antibody. LC3-II/LC3-I and LC3-II/Vinculin ratios are increased in Torin-treated cells. (**D)** Western blotting of protein lysates from untreated, Ad-null, TFEB-, and TFEBmt-treated myotubes shows a significant increase in the levels of phospho-ERK in TFEB-treated cells. Vinculin was used as a loading control. Note that the ERK1/2 kinase runs as two bands of 42 and 44 kDa. At least three experiments were performed for each condition.

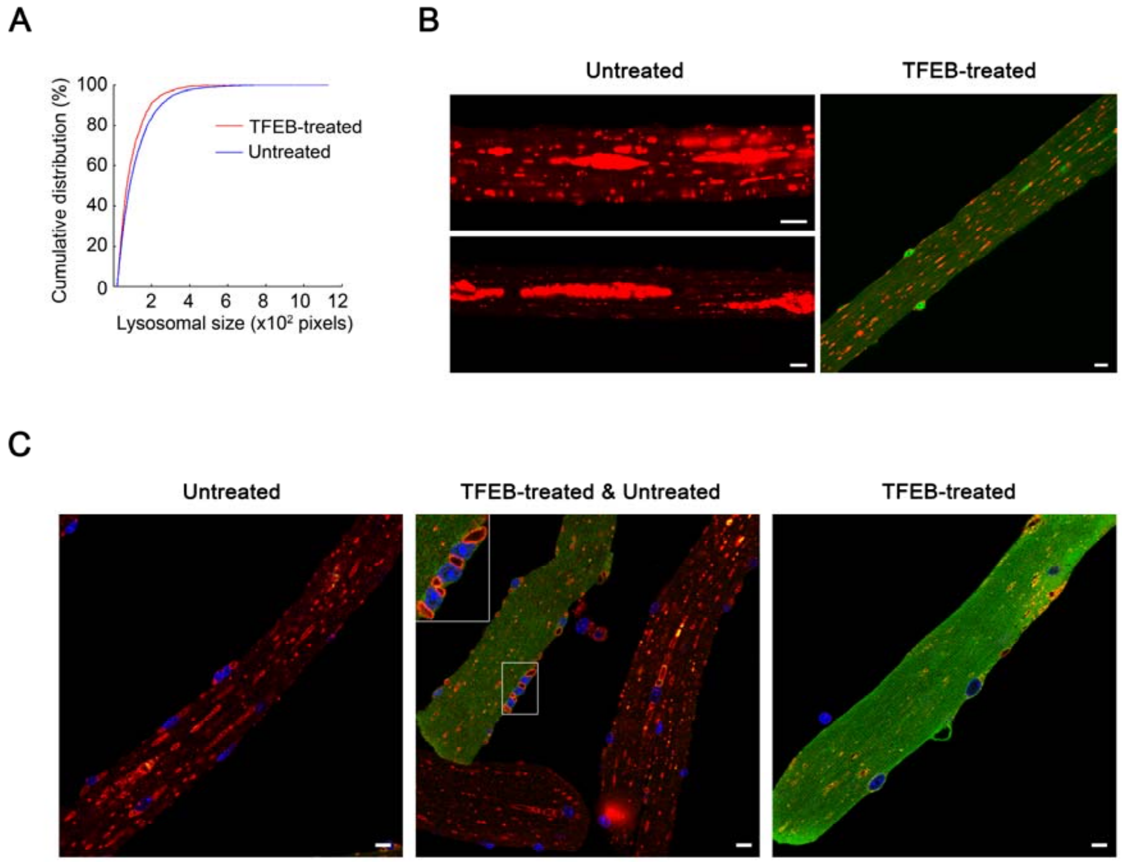

Figure S6. TFEB promotes clearance of enlarged lysosomes in PD fibers.

(A) Distribution of lysosomal size in untreated and GFP-TFEB-treated muscle fibers ($p=1.12x10^{-13}$; n=10 fibers for each condition). (**B**) Confocal microscopy images of live fibers derived from 3 month-old untreated (left) or GFP-TFEB-treated (right) GAA-/- mice. The fibers were stained with LysoTracker®. (**C**) Confocal microscopy images of fixed fibers derived from 3 month-old untreated (left and middle) or GFP-TFEB-treated (middle and right) GAA-/- mice. The fibers were stained with LAMP1. The effects of TFEB are clearly visible – reduction in lysosomal size, appearance of normal size lysosomes, and lysosomal docking to the plasma membrane (inset). Bar: 10 µm.

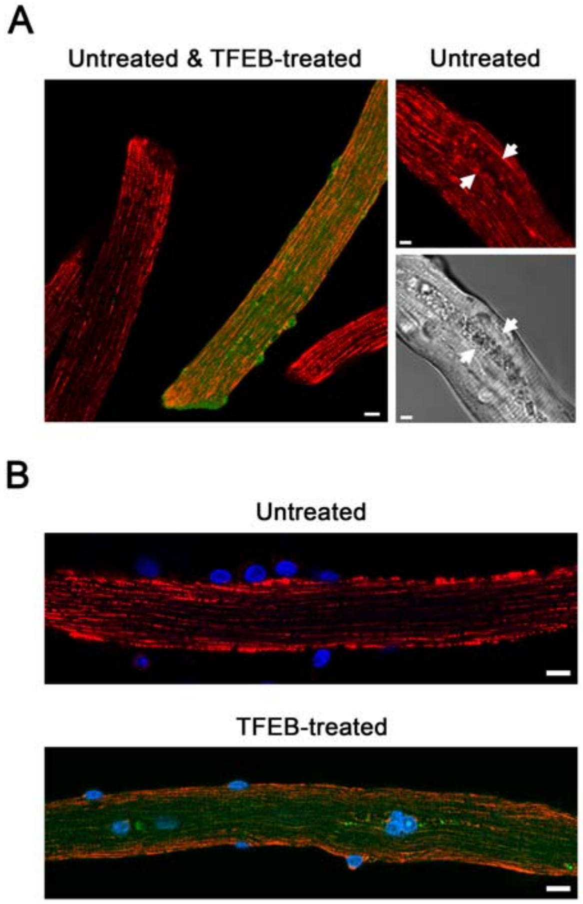

Figure S7. TFEB expression in PD fibers does not affect mitochondrial distribution and abundance.

Confocal microscopy images of live (**A**) or fixed (**B**) fibers derived from 3 month-old untreated or GFP-TFEB-treated GAA-/- mice. Live fibers were stained using the mitochondrial-specific dye MitoTracker Red; fixed fibers were immunostained with anti-cytochrome c antibody. The distribution pattern and abundance of mitochondria appear similar in TFEB-treated and untreated muscle fibers. MitoTracker staining is suitable for detecting major mitochondrial abnormalities as shown by the absence of the dye in the area of autophagic buildup (right panels, white arrows in **A**).

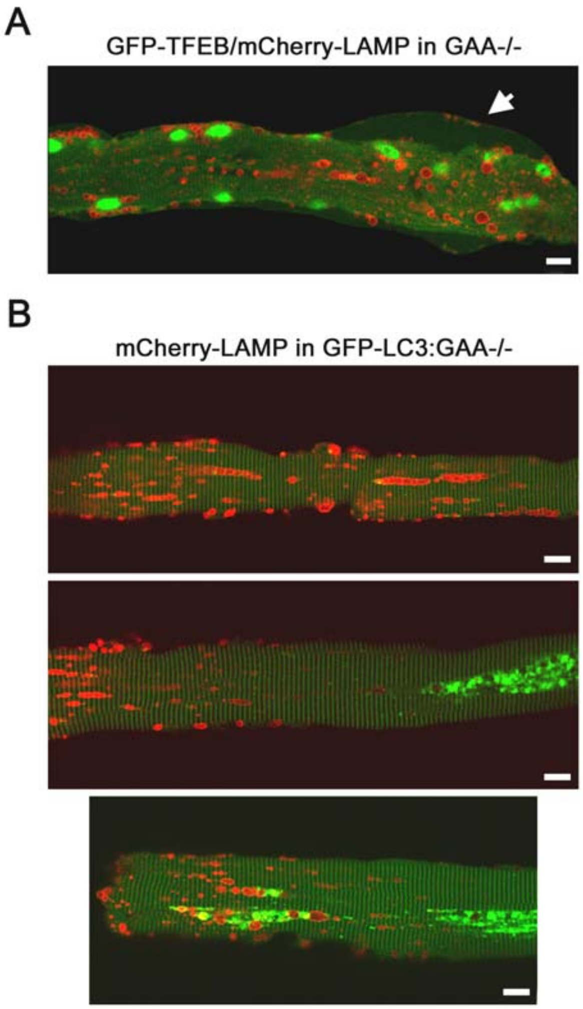

Figure S8. (A) Occasional TFEB- transfected fibers exhibit membrane detachment. Lysosomes are red; TFEB (green) is localized primarily in nuclei. Membrane blebbing in TFEB-expressing cell may result from massive expulsion of the lysosomes. (**B**) **Different patterns of mCherry-LAMP expression in FDB fibers from GFP-LC3:GAA-/- mice.** The distribution of labeled lysosomes (red) and autophagosomes (green) in the majority of the transfected fibers is mutually exclusive: expression of LAMP is predominantly seen in fibers (top) or a portion of a fiber (middle) free from autophagic buildup. Labeled lysosomes and autophagosomes are seen in close proximity in 10-15% of transfected fibers. Bar: 10 µm.

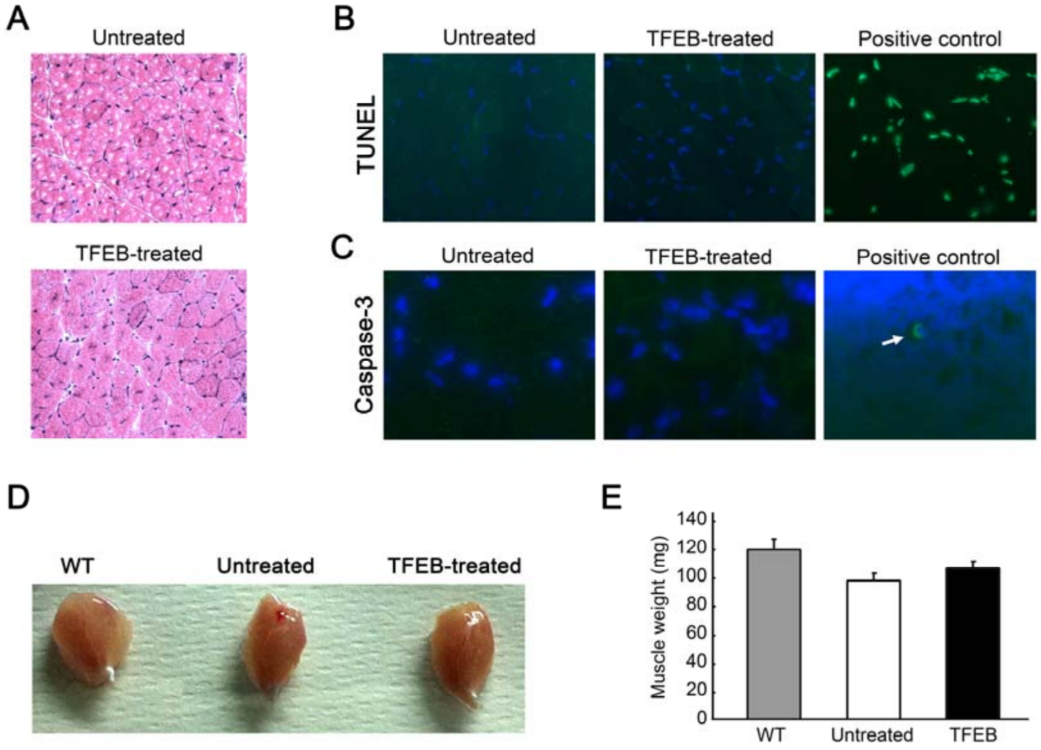

Figure S9. TFEB expression does not result in any appreciable abnormalities in PD muscle.

AAV2/1-TFEB was injected into the gastrocnemius muscle of one month-old GAA-/- mice; muscles were analysed 45 days after. **(A)** H&E staining of untreated and TFEB-treated muscle. (**B**) Cryosections of muscle biopsies stained by TUNEL. The section treated with DNAse I to fragment DNA served as a positive control. (**C**) Cryosections of muscle biopsies immunostained with anti-Caspase-3 antibody. A cryosection of P3 mouse retina served as a positive control. (**D**) Representative images of muscle preparations. (**E**) Muscle weights expressed in mg.

Table S1. Acid phosphatase assay in non-treated and Ad-TFEB-treated myotubes*

**condition**	**p-nitrophenol (µM)** (mean ± SD)
non-treated n=4	0.0877 ± 0.0237
TFEB-treated n=4	**0.1659 ± 0.0500

* the assay was performed in culture medium from non-treated and TFEB-treated muscle cells; the medium was incubated with substrate buffer [0.5 M NaAcetate, pH 4.4, 0.5% Triton X-100, 5 mM 4-Nitrophenyl phosphate disodium (Sigma-Aldrich)] for 1 h at 37° C; following the addition of stop buffer (0.5 M glycine, 0.5 M Na_2CO_3, pH 10), the resulting product, p-nitrophenol, was measured at 405 nm. P-nitrophenol solution (Sigma-Aldrich) was used for the standard curve, and acid phosphatase (Sigma-Aldrich) was used as a positive control.

** $P<0.01$

Equipment and settings

Images were acquired on a Zeiss LSM 510 confocal microscope using a 63x 1.40 NA oil objective. Excitation and emission setting were as follows: GFP/Alexa 488: MBS 488, excitation 488 nm, emission, BP 505-550 nm; mCherry/Alexa 568, MBS 488/543, excitation, 543 nm, emission, LP 560 nm; Hoechst: MBS 405, excitation, 405 nm, emission, BP 420-480. All images were collected sequentially by color by using multiple tracks, at 8 bit depth and 1 AU pinhole settings.

Additional Methods

Generation and genotyping of mice. GFP-LC3 transgenic mice [generated by Dr. Mizushima (Mizushima et al, 2004) and distributed by the RIKEN Bio-Resource Center (Japan)], were crossed with GAA-/- mice to produce GFP-LC3:GAA-/- mice. Transgenic mice expressing the temperature-sensitive SV40 large T antigen tsA58 under the control of the interferon-inducible murine H-2K^b promoter [Immortomouse®; CBA;B10-Tg(H-2K^b-tsA58)6Kio/Crl] were obtained from Charles River Laboratories (Wilmington, MA). These mice were crossed with GAA-/- mice (C57BL/6/129/Sv inbred strain)(Raben et al, 1998) to produce (H-2K^b-tsA58)+/-:GAA-/- (referred to as immortoGAA-/-) or with GFP-LC3:GAA-/- mice to produce (H-2K^b-tsA58)+/-:GFP-LC3:GAA-/- (referred to as immorto:GFP-LC3:GAA-/-) mice. Genomic DNA was isolated from tail clips using Quickgene DNA Tissue Kit (FujiFilm Life Sciences, Tokyo, Japan) according the manufacturer's instructions. The presence of the H-2K^b-tsA58 and GFP-LC3 transgenes was detected as described (Jat et al, 1991; Kern & Flucher, 2005; Mizushima et al, 2004). The *GAA* wild type, *GAA +/-*, and *GAA-/-* alleles were detected with a combination of three primers: tcctgagcccaaacacttctt (GAA6F)/cctacccacaacatccaggtatt (GAA6R)/ctgacaccagaccaactggtaat (neoR). The presence of a wt allele is indicated by a ~150 bp and, whereas the presence of a mutant allele is indicated by a ~ 450 bp product.

Isolation of live fibers. The dissected FDB muscle was digested in 0.2% (w/v) collagenase type 1 (Sigma-Aldrich) in Dulbecco's Modified Eagle Medium (DMEM) (Life Technologies) with 0.1% (w/v) BSA for 2 hours at 37°C in an atmosphere of 5% CO2 with gentle shaking. The muscle was then transferred to DMEM containing 10% Fetal Bovine Serum (FBS) (Atlanta Biologicals, Lawrenceville, GA, USA) and 1X Penicillin/Streptomycin (P/S)/L-Glutamine (Life Technologies). Individual fibers were released under a dissecting microscope and plated on BD Matrigel (BD Biosciences, San Jose, CA)-coated ibiTreat µ–Slide 8-well chambers or µ-Dishes 35 mm (for time-lapse experiments) (ibidi GmbH, Martinsried, Germany ibiTreat µ–Slide). The plating DMEM medium (high glucose) contained 10% horse serum (HS) (Thermo Fisher, Rockford, IL), 0.5% chick embryo extract (Sera Lab, West Sussex, UK), 10mM HEPES, and 1X P/S/L-Glutamine. Under this condition the fibers can survive in culture for several days

Post-acquisition image processing and analysis. To determine the cumulative distribution of lysosomal size in myotubes, confocal images were processed with ImageJ software (1): color channels were split to isolate LAMP-stained vesicles, images were converted to 8-bit format, thresholds were manually adjusted to depict complete vacuolar structures with minimal over-

saturation, and particle analysis (size: 200-3000, circularity: 0.00-1.00, "fill holes" selected) was performed to provide pixel values of lysosomal vacuolar areas. Cumulative % values were calculated as individual pixel areas divided by the total number of lysosomes per condition (for myotubes, n=703 for Ad-TFEB infected, n=1395 for Ad-Null infected). Since ImageJ software is not well suited for differentiating between individual enlarged lysosomes within clusters, we employed CellProfiler cell image analysis software for the evaluation of lysosomal size in myofibers. Ten fibers were analyzed for each condition and cumulative % values were calculated as above (n=1138 lysosomes for TFEB-treated fibers; n= 1558 lysosomes for untreated fibers). Distributions were compared by the two-sample Kolmogorov-Smirnov test and distribution medians were compared by the Wilcoxon rank-sum test for equal medians.

Maximal lysosomal velocities were calculated from time-lapse confocal image series with ImageJ software and the Manual Tracking plug-in (parameters: x, y=0.44μm, t=10 min) (1, 2). To minimize the contribution of fiber movement to lysosomal velocity measurements, manual tracking was performed only between time points when fiber twitching was not ostensible. Three to six fibers were analyzed per condition, and up to 10 lysosomes per fiber were selected on the basis of their apparent motility (i.e., lysosomes exhibiting no discernible movement were excluded from consideration). For each condition, the pooled values of the three highest velocities per lysosome were averaged to obtain the maximal lysosomal velocity, and 95% confidence intervals were calculated (error bars; 1.96*SE). For all statistical analyses, Student's t-test calculations were performed in Excel.

The number of large lysosomes per fiber area was calculated in LSM Image Browser. In single confocal image planes, the maximum diameter of each lysosome was measured. The number of lysosomes equal to or exceeding 3.5 μm in length was recorded and divided by total fiber area.

For EM data, quantification of the number of lysosome-like organelles and their dimensions as well as the number of autophagosomes was performed using the iTEM software (Soft Imaging Systems GmbH, Munster, Germany).

For all statistical analyses, Student's t-tests and 95% confidence intervals (error bars; 1.96*SE) were calculated in Excel.

Movie captions

Movie 1. GFP-TFEB- and mCherry-LAMP-transfected fiber derived from a GAA-/- mouse.

Movie 2. mCherry-LAMP-transfected fiber derived from a GFP-LC3:GAA-/- mouse.

Movie 3. Membrane detachment in a GFP-TFEB- and mCherry-LAMP-transfected fiber derived from a GAA-/- mouse.

Movie 4. Autophagic buildup in a mCherry-LAMP-transfected fiber derived from a GFP-LC3:GAA-/- mouse.

Movie 5. Surge in autophagy in a mCherry-LAMP-transfected fiber derived from a GFP-LC3:GAA-/- mouse.

Movie 6. Surge in autophagy in a Flag-TFEB- and mCherry-LAMP-transfected fiber derived from a GFP-LC3:GAA-/- mouse.

Movie 7. Docking of autolysosomes to the plasma membrane in a Flag-TFEB- and mCherry-LAMP-transfected fiber derived from a GFP-LC3:GAA-/- mouse.

Movie 8. GFP-TFEB- and mCherry-LAMP-transfected fiber derived from an autophagy-deficient GAA-/- mouse (Atg7:GAA DKO).

References:

Jat PS, Noble MD, Ataliotis P, Tanaka Y, Yannoutsos N, Larsen L, Kioussis D (1991) Direct derivation of conditionally immortal cell lines from an H-2Kb-tsA58 transgenic mouse. Proc Natl Acad Sci U S A 88: 5096-5100

Kern G, Flucher BE (2005) Localization of transgenes and genotyping of H-2kb-tsA58 transgenic mice. Biotechniques 38: 38, 40-42

Mizushima N, Yamamoto A, Matsui M, Yoshimori T, Ohsumi Y (2004) In vivo analysis of autophagy in response to nutrient starvation using transgenic mice expressing a fluorescent autophagosome marker. MolBiolCell 15: 1101-1111

Raben N, Nagaraju K, Lee E, Kessler P, Byrne B, Lee L, LaMarca M, King C, Ward J, Sauer B, Plotz P (1998) Targeted disruption of the acid alpha-glucosidase gene in mice causes an illness with critical features of both infantile and adult human glycogen storage disease type II. JBiolChem 273: 19086-19092

Raben N, Ralston E, Chien YH, Baum R, Schreiner C, Hwu WL, Zaal KJ, Plotz PH (2010a) Differences in the predominance of lysosomal and autophagic pathologies between infants and adults with Pompe disease: implications for therapy. Mol Genet Metab 101: 324-331

Thurberg BL, Lynch MC, Vaccaro C, Afonso K, Tsai AC, Bossen E, Kishnani PS, O'Callaghan M (2006) Characterization of pre- and post-treatment pathology after enzyme replacement therapy for pompe disease. Lab Invest 86: 1208-1220

Software references:

Rasband, W.S., ImageJ, U. S. National Institutes of Health, Bethesda, Maryland, USA, http://imagej.nih.gov/ij/, 1997-2012.

Cordelieres, F., Manual Tracking Plug-in, Institut Curie, Orsay, France, http://rsbweb.nih.gov/ij/plugins/track/track.html, 2004-2012.
